# Nanoplastics Enhance Transmembrane Transport and Uptake of Carcinogens: Transcriptional Changes and the Effects of Weathering

**DOI:** 10.1002/advs.202507355

**Published:** 2025-06-20

**Authors:** Erik B. Schiferle, Saatwik Suman, Katherine R. Steffen, Koustav Kundu, Aniqa N. Islam, Björn M. Reinhard

**Affiliations:** ^1^ Division of Materials Science and Engineering Department of Chemistry and The Photonics Center Boston University Boston MA 02215 USA; ^2^ Department of Chemistry and The Photonics Center Boston University Boston MA 02215 USA

**Keywords:** intestinal membrane, nanoplastics, polycyclic aromatic hydrocarbons (PAHs), RNA, transmembrane transport

## Abstract

Nanoplastics are generated from common consumer plastics (polyethylene terephthalate, high‐density polyethylene, polystyrene, polyvinyl chloride) and exposed to simulated marine weathering for up to 10 weeks. Fourier‐transform infrared spectroscopy and ζ‐potential measurements reveal continuous changes in the composition of the nanoplastics, consistent with oxidation. Although the chemical composition and oxidation of the nanoplastics influence their ability to sorb polycyclic aromatic hydrocarbons (PAHs), for all investigated conditions, sorption of PAHs to nanoplastics achieves effective PAH concentrations that are orders of magnitude higher than the solubility limit in water. In an intestinal co‐culture model membrane consisting of M cells and enterocytes, PAH‐loaded nanoplastics enhance the overall PAH transport into and across the membrane, with HDPE achieving the highest intracellular PAH concentration. RNA sequencing of cell membranes exposed to nanoplastics reveals significant transcriptional changes, including upregulation of oxidative stress and detoxification pathways (NQO1, CYP1A1, CYP1B1), especially in response to PAH‐loaded nanoplastics, while genes associated with basic cell functions, such as DNA repair (MACROD2) and division (KIF20A), are downregulated. These findings confirm the feasibility of nanoplastics to increase bioaccessibility and bioavailability of hydrophobic carcinogens and enhance cellular stress, which underscores the potential environmental and health impacts associated with nanoplastics as carriers of hydrophobic environmental toxins.

## Graphical abstract

Nanoplastics exposed to defined weathering conditions are systematically investigated as vectors for polycyclic aromatic hydrocarbons (PAHs). This investigation demonstrates significant potential for enhancing PAH uptake and transport in an intestinal membrane model. Furthermore, cells exposed to nanoplastics reveal characteristic transcriptional changes as function of weathering state and PAH loading.

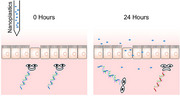

## 1 Introduction

The pervasive use of plastics in consumer products and as packaging material results in the release of large quantities of plastics into the environment, much of it into aquatic ecosystems. It has been estimated that 109 million metric tons of plastic waste have accumulated in rivers as of 2019^[^ ^1^ ^]^ and that an estimated total of ≈30 million metric tons has been released into the oceans, with an additional annual increase between 1.7 and 8 million metric tons.^[^ ^1^, ^2^ ^]^ Due to the longevity of petroleum‐derived plastics, this scale of pollution has made plastic debris ubiquitous in aquatic ecosystems. Plastic pollution is not limited to macroscopic debris such as bags, bottles, and other trash. Rather, these macroscopic plastic objects degrade or “weather” into smaller particles when exposed to the elements, ultimately reaching micro‐ to nanometer size scales.^[^ ^3^, ^4^ ^]^ Plastic particles with dimensions between 1 µm and 5 mm are commonly referred to as microplastics, and even smaller particles with dimensions <1 µm are designated as nanoplastics.^[^ ^5^, ^6^ ^]^ The weathering rate of plastics waste depends, in addition to environmental conditions, on its chemical composition as well as its size and morphology.^[^ ^7^, ^8^ ^]^ Release of nanoplastics into the oceans is of concern not only because the small size and slow degradation of the particles creates risks of a systemic distribution in marine organisms after uptake, potentially resulting in the penetration of organs, tissues and single cells,^[^ ^9^, ^10^ ^]^ but also because humans are ultimately exposed to the plastics pollution through the food chain.^[^ ^11^, ^12^ ^]^

Ingested micro‐ and nanoparticles can be absorbed in the intestinal barrier of the small intestine by transcytosis through enterocytes and M cells^[^ ^13^, ^14^, ^15^ ^]^ or by paracellular transport, more typical of very small particles or molecules.^[^ ^16^, ^17^ ^]^ Interactions between plastic particles and intestinal epithelial cells can cause cell damage, intestinal membrane inflammation, and eventually reduce intestinal barrier functions.^[^ ^18^, ^19^ ^]^ The specific toxicity associated with micro‐ and nanoplastics at the intestinal membrane is expected to depend on the size, shape, and composition of the particles. But, cell stress^[^ ^20^, ^21^ ^]^ and metabolic changes^[^ ^22^, ^23^ ^]^ are frequently observed responses to plastic particles.^[^ ^24^ ^]^ In addition to the outlined “direct” risks associated with ingested plastics particles, additional risks derive from the potential of plastics particles to serve as vector of hydrophobic, hazardous compounds.^[^ ^25^, ^26^ ^]^ Due to their large surface‐to‐volume ratio, nanoplastics, in particular, have a large capacity for sorbing payload and are therefore of significant concern. Although the surface properties of nanoplastics undergo changes in the course of the environmental weathering process, it was demonstrated previously that nanoplastics retain their ability to sorb hydrophobic compounds, such as polycyclic aromatic hydrocarbons (PAHs), under simulated weathering conditions.^[^ ^27^ ^]^

PAHs are carcinogenic persistent organic pollutants (POPs) that are generated by incomplete oxidation of organic materials in either anthropogenic or natural combustion processes.^[^ ^28^ ^]^ Due to their very low solubility in water, PAHs are sequestered into sediment or associated with dispersed organic matter in aquatic ecosystems.^[^ ^29^ ^]^ Given the ever‐increasing pollution of aquatic systems with plastics, the ability of nanoplastics to sorb PAHs raises concerns about an increased mobilization of PAHs.^[^ ^27^ ^]^ Indeed, micro‐ and nanoplastics have been demonstrated to yield a significant increase of POPs in seawater and orders of magnitude higher bioaccumulation of PAHs and other hydrophobic organic chemicals in tissue.^[^ ^30^, ^31^, ^32^ ^]^ A higher effective PAH concentration in aquatic ecosystems due to nanoplastics pollution would increase the risk of human exposure through contaminated food or water, which is problematic since long‐term exposure to PAHs has been linked to a variety of adverse health effects and diseases, including cancer, developmental abnormalities, and neurological deficits in humans.^[^ ^33^, ^34^, ^35^ ^]^

Although prior work has provided evidence that nanoplastics sorb PAHs and increase both bioaccessibility and bioavailability,^[^ ^32^, ^36^, ^37^, ^38^, ^39^, ^40^ ^]^ the mechanisms underlying a nanoplastics‐enhanced PAH transport across the intestinal membrane and the effect of nanoplastics weathering remain unclear. One concern is that stress as result of nanoplastics exposure in enterocytes and M cells decreases intestinal barrier functions and, thus, results in increased membrane permeability for PAHs.^[^ ^18^, ^19^ ^]^ Synergistic effects between PAHs and nanoplastics in inducing cell stress and increasing transepithelial PAH transport are conceivable and require characterization. The small intestine plays a key role not only for the absorption of PAHs associated with ingested nanoplastics but also their metabolization.^[^ ^41^, ^42^, ^43^ ^]^ We chose an in vitro intestinal membrane model as the platform in this study to systematically evaluate how nanoplastics alone and in combination with sorbed PAHs affect intestinal cells and transepithelial transport. Specifically, we set out to i) quantify the capacity of nanoplastics to sorb PAHs and the extent to which the PAH‐loaded nanoplastics mediate transport of PAHs into and across the model membrane, and ii) characterize the effect of nanoplastics, with and without PAH “payload,” on gene expression as a measure of the cellular response to these environmental toxins. The underlying processes depend on the sorption and release properties of PAHs to/from nanoplastics. These properties can be affected by size, morphology, and chemical composition, which change during the weathering of plastics in the environment. Consequently, the experiments of this study were performed with common consumer plastics composed of polyethylene terephthalate (PET), high‐density polyethylene (HDPE), polystyrene (PS), and polyvinyl chloride (PVC) before and after accelerated weathering. The effect of the weathering process on the chemical structure of the plastics and its impact on PAH sorption are evaluated.

## 2 Results

### 2.1 Nanoplastics Preparation, Size & Charge Determination, and Vibrational Characterization

**Figure 1:**
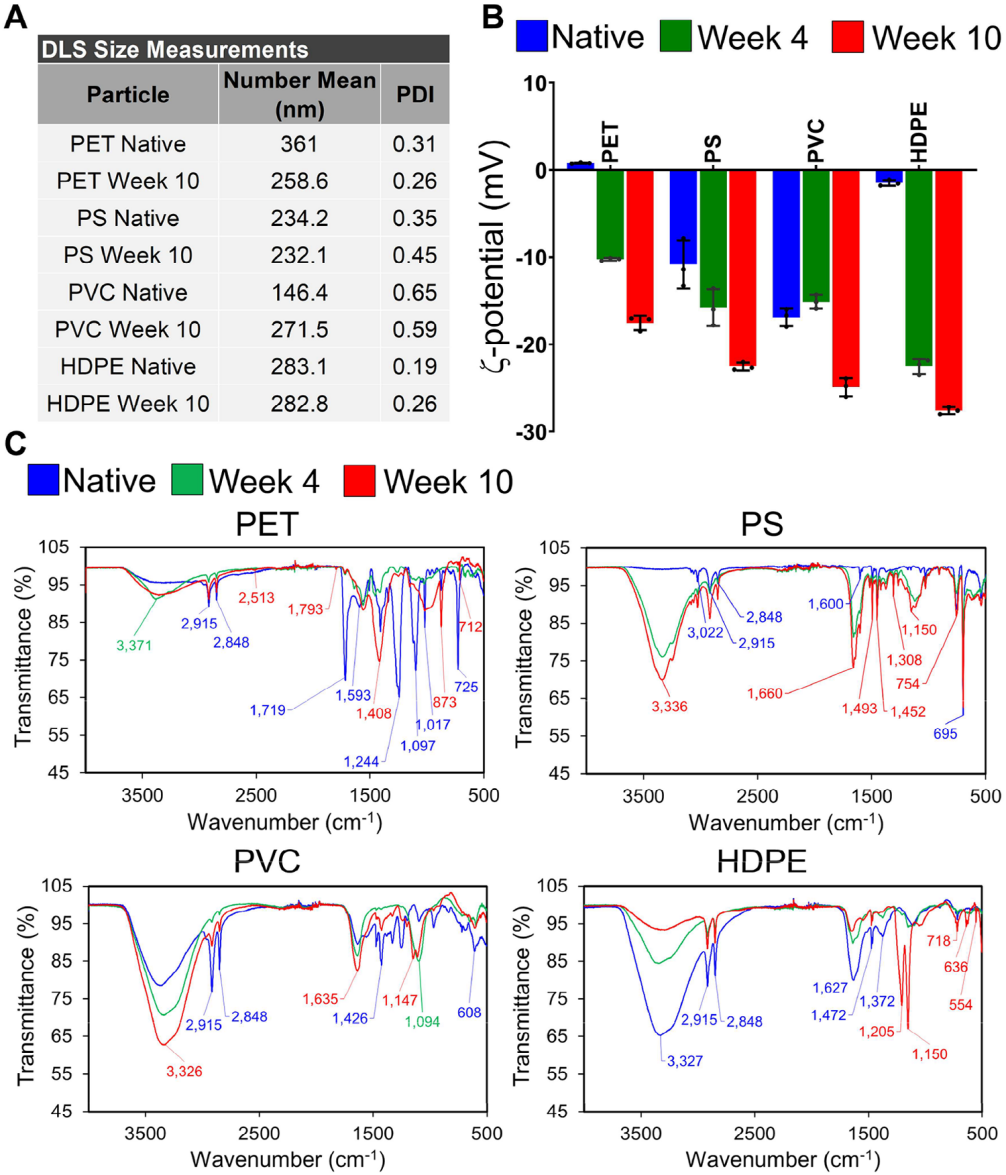
Nanoplastics characterization. A) Number mean (nm) and polydispersity index (PDI) of the hydrodynamic diameter (nm) determined by dynamic light scattering (DLS) for native and 10 weeks weathered nanoplastics. B) ζ‐potentials (mV) for native and weathered (week 4, 10) nanoplastics. Error bars represent the mean ζ‐potential ± standard deviation (SD, *n* = 3 repeat measurements). C) Fourier‐transform infrared (FTIR) spectra for PET, PS, PVC, and HDPE nanoplastics with different degrees of weathering (native, week 4, and week 10).

Nanosized plastics were generated following an established procedure (Figure S1A,B, Supporting Information).^[^ ^27^ ^]^ Briefly, consumer plastic objects representing a distribution of widely used plastics in the environment were blended and milled independently. Designated by the recycling stamp on the macroscopic samples, plastics composed of PET, HDPE, PS, and PVC plastics were included in this work. After milling, the generated nanoplastic particles, termed “native” nanoplastics, underwent simulated marine environmental conditions, consisting of mechanical agitation in seawater and photochemical weathering using a commercial sunning‐lamp emitting UV‐A and UV‐B light. The nanoplastics were exposed to weathering for a total of 10 weeks, with periodic sampling at weeks 0 (native), 1, 2, 3, 4, 5, 8, and 10, to enable a continuous assessment of the weathering progress. The hydrodynamic diameters of the nanoplastics were determined by dynamic light scattering (DLS) (**Figures** **1**A and S1A, Supporting Information). The experiments show composition‐dependent differences, with native PET nanoplastics having the largest diameter of ≈361 nm and native PVC nanoplastics the smallest of ≈146 nm. The changes in size associated with photochemical weathering for HDPE and PS nanoplastics were minimal, while PET nanoplastics decreased ≈28% and PVC nanoplastics increased ≈86%. The surface charges of nanoplastics particles suspended in deionized (DI) water^[^ ^27^ ^]^ were characterized through ζ‐potential measurements (Figure 1B). For all samples the ζ‐potentials become more negative as a result of the photochemical weathering from UV light exposure. Both observations are consistent with the materials being oxidized as a function of time. The changes are particularly prominent for PET and HDPE, which have ζ‐potentials close to 0 in the native state.

To characterize the molecular changes associated with photochemical weathering, we recorded vibrational spectra through Fourier‐transform infrared (FTIR) spectroscopy (Figure 1C). We recorded FTIR spectra of PET, HDPE, PS, and PVC nanoplastics after weeks 0 (native), 1, 2, 3, 4, 5, 8, and 10 of photochemical weathering (native, week 4, and week 10 are shown). For PET, the broad band at ≈3371 cm^−1^ in the spectrum of the 0 weeks weathering (native) nanoplastics indicates the presence of hydroxyl (OH) groups, likely from surface oxidation or contamination, potentially from recycling.^[^ ^44^ ^]^ The bands at 2915 and 2848 cm^−1^ are assigned to the asymmetric and symmetric CH stretching modes of the aliphatic segments of the main chain,^[^ ^45^ ^]^ while the 1719 cm^−1^ band marks carbonyl groups in PET.^[^ ^46^, ^47^, ^48^ ^]^ The benzene C═C stretching mode of PET is located at 1593 cm^−1^, and the bands at 1244 and 1097 cm^−1^ lie in the range of ester stretching modes along the PET chain. The feature at 725 cm^−1^ is linked to the C─H wagging mode on the para‐functionalized aromatic ring of PET.^[^ ^46^, ^47^, ^48^ ^]^ After 4 weeks of weathering, the spectra still contain these bands, but most features, except for the OH band at 3371 cm^−1^ have decreased in intensity. The 10‐week weathered spectra reveal new distinct bands between 1400 and 1500 cm^−1^, as well as at 712, 873, 1793, and 2513 cm^−1^. These bands suggest calcium carbonate formation,^[^ ^27^, ^49^ ^]^ and align with a degradation process in which PET photo‐oxidation under oxygen‐rich conditions releases CO_2_, leading to CaCO_3_ deposition in the presence of Ca^2+^ ions in seawater.^[^ ^27^, ^50^, ^51^ ^]^

In the case of the native PS nanoplastics spectrum, the band at 3022 cm^−1^ is assigned to the stretching mode of aromatic C─H bonds.^[^ ^52^, ^53^ ^]^ The bands at 2915, 2848, and 1452 cm^−1^ correspond to C─H stretching (asymmetric and symmetric) as well as bending modes of aliphatic groups in the backbone chain, respectively.^[^ ^52^ ^]^ C═C and C─C stretching vibrations associated with the aromatic ring in PS are detected at 1600 and 1493 cm^−1^, respectively,^[^ ^52^, ^53^ ^]^ and the features at 754 and 695 cm^−1^ are indicative of C─H out‐of‐plane bending modes of the aromatic ring.^[^ ^52^, ^53^, ^54^ ^]^ Photochemical weathering induces new broad features at 1660, 1150, and 3336 cm^−1^, which are indicative of PS oxidation. The peak at 1660 cm^−1^ may be assigned to a C═C stretching mode, a carbonyl C═O stretching mode, or a combination of both. The 1150 cm^−1^ peak is associated with C─O─R stretching modes of ester groups (R‐COO‐R′), ether groups (R‐O‐R′), or alcohols. The band at 3336 cm^−1^ is characteristic of OH groups. Based on the recorded spectral data, a degradation process that results in the formation of C═C groups (e.g., in the form of styrene fragments) and secondary alcohols seems likely.

In the FTIR spectrum of the PVC nanoplastics sample, bands at 2915 and 2848 cm^−1^ again correspond to the asymmetric and symmetric stretching modes of the CH groups in the polymer backbone.^[^ ^52^ ^]^ The pronounced vibrational feature at 608 cm^−1^ and weaker features in the range up to 700 cm^−1^ are characteristic absorption peaks of C─Cl stretching vibrations.^[^ ^47^, ^52^ ^]^ Spectral features between 1255 and 1426 cm^−1^ are associated with CH_2_ wagging, scissoring, and C─H (H─C─Cl) bending modes.^[^ ^55^ ^]^ We also observe features at 3400–3500, 1635, and 1500–1600 cm^−1^, which cannot be assigned to PVC and may indicate the presence of hydroxyl, and C═C or amine groups from additives, such as plasticizers, impact modifiers and stabilizers. Double bonds in the backbone of PVC can also result from dehydrochlorination.^[^ ^56^, ^57^ ^]^ Exposure to simulated weathering conditions results in an increase in spectral intensity between 3400 and 3500 cm^−1^ as well as at 1635 cm^−1^, a decrease in intensity between 1500 and 1600 cm^−1^ and at 1426 cm^−1^, and the emergence of new bands at 1094 and 1147 cm^−1^ by week 10. The new bands could be indicative of several different types of bonds or functional groups. C─O─R stretching modes in alcohols, ethers,^[^ ^58^ ^]^ carboxylic acids, and esters fall within this range.^[^ ^59^, ^60^ ^]^ However, due to a lack of a characteristic ester or carboxylic acid carbonyl peak between 1700 and 1730 cm^−1^ and an increase in the OH associated signal in the range between 3400 and 3500 cm^−1^, the formation of alcohols seems more plausible. The increase at 1635 cm^−1^ indicates the formation of unconjugated C═C groups, presumably formed by dehydrochlorination, which is also consistent with a decrease in CH_2_ signal between 1255 and 1426 cm^−1^.

In the case of the HDPE sample, the spectra contain symmetric and asymmetric stretching modes of CH groups in the polymer backbone at 2848 and 2915 cm^−1^, respectively,^[^ ^27^, ^52^, ^53^ ^]^ and these features remain relatively constant over time during photochemical weathering. The band at 1472 cm^−1^ is assigned to the CH_2_ asymmetric in‐plane bending/scissor mode of the HDPE chain.^[^ ^52^, ^53^ ^]^ The 1372 cm^−1^ band originates from the symmetric wagging (bending) mode of CH_2_ units,^[^ ^61^, ^62^ ^]^ and the 718 cm^−1^ feature corresponds to the rocking mode of CH_2_.^[^ ^52^, ^53^, ^58^ ^]^ Atypical bands are also seen at 1627 and 3327 cm^−1^, which suggests additives, plasticizers, contaminants or some degree of oxidation. The band at 1627 cm^−1^ typically falls in the range of C═C or C═O bonds, while the band at 3327 cm^−1^ indicates the presence of OH groups. The HDPE sample was harvested from a white milk jug, which typically contains titanium dioxide for coloring and UV protection. These metal oxide nanoparticles can contribute to the measured FTIR spectrum by adding their own FTIR absorption bands as well as by trapping other molecules such as water, which absorbs at 1627 and 3327 cm^−1^ wavenumbers.^[^ ^63^, ^64^ ^]^ Photochemical weathering under the chosen conditions changes the polymer. A decrease in OH associated signal at 3327 cm^−1^, emergence of a strong feature with peaks at 1150 and 1205 cm^−1^, and additional features at 636 and 554 cm^−1^ are observed after weathering. The 1150 and 1205 cm^−1^ peaks are typical of C─O─R stretching modes, which would suggest formation of alcohols, esters, or ethers. However, due to the decrease in signal intensity of the OH associated signal at 3327 cm^−1^ as well as in the C═O region, the formation of ethers during the photo‐oxidation of HDPE seems more likely. Intriguingly, the color of the sample turned green after 10 weeks of weathering, which may indicate that metal ions from seawater are interacting with the HDPE matrix or degradation products and that the signals at 636 and 554 cm^−1^ arise from metal‐oxygen stretching or bending vibrations.

Overall, the FTIR measurements are in general agreement with the compositional assignments of the recycling stamps. All plastics show indications of oxidation, which is consistent with the ζ‐potentials becoming more negative (Figure 1B). For PET, PS, and PVC distinct spectral markers of photodegradation were detected in response to weathering. The PET spectra indicate deposition of calcium carbonate in response to CO_2_ release through weathering, while PS exhibits signs of C═C removal, secondary alcohol formation, and/or carbonyl formation. The spectra of weathered PVC contain features characteristic of C═C groups and different oxidized carbon species. In the case of HDPE, weathering induces spectral changes indicative of ether bonds and metal ion stabilized oxides. However, the HDPE spectra also contain evidence of significant changes associated with additives or contaminants, which complicates the interpretation of the spectra. To validate the oxidation indicated by the recorded FTIR spectra, we performed X‐ray photoelectron spectroscopy (XPS) on native nanoplastics as well as for week 10 weathered nanoplastics of PET and PS. In both cases, C1s scans illustrate oxidation (C═O, C─O) induced by weathering (Figure S2A,B, Supporting Information).

### 2.2 PAH Sorption

**Figure 2:**
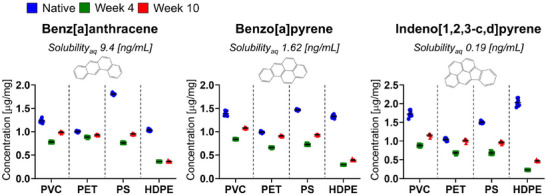
Quantification of PAH sorption. PAH concentration in µg per mg of nanoplastics for PVC, PET, PS, HDPE nanoplastics weathered for 0 weeks (native), 4 weeks, or 10 weeks. Error bars represent the mean ± SD (*n* = 8 replicates).

We characterized the loading of the PAHs benz[a]anthracene (molecular weight, MW = 228 g mol^−1^), benzo[a]pyrene (MW = 252 g mol^−1^), and indeno[1,2,3‐c,d]pyrene (MW = 276 g mol^−1^) onto nanoplastics using an accelerated loading protocol, which reduces the loading time from typically several months in the case of passive accumulation of PAHs on nanoplastics in water, to several days without inducing detectable chemical or morphological changes.^[^ ^27^ ^]^ Specifically, we measured the concentration of the three selected PAHs sorbed onto nanoplastics composed of PVC, PET, PS, or HDPE, for native as well as for weathering of plastics for 4 and 10 weeks under simulated weathering conditions (**Figure** **2** and Table S1, Supporting Information). For benz[a]anthracene, concentrations decrease from the native condition to week 4 and then slightly increase by week 10. For instance, PVC shows a concentration (mass PAH per mass nanoplastics) drop from 1.233 ± 0.041 µg mg^−1^ for native PVC to 0.779 ± 0.012 µg mg^−1^ in week 4, then a rise to 0.991 ± 0.014 µg mg^−1^ in week 10. Similar trends are observed for PET, PS, and HDPE. For benzo[a]pyrene the loading of PVC starts at 1.388 ± 0.048 µg mg^−1^ in the native condition, drops to 0.840 ± 0.01 µg mg^−1^ in week 4, and then rises to 1.081 ± 0.012 µg mg^−1^ in week 10. PET, PS, and HDPE nanoplastics behave similarly. For indeno[1,2,3‐c,d]pyrene, native PVC nanoplastics shows a loaded concentration of 1.724 ± 0.087 µg mg^−1^, which decreases to 0.885 ± 0.024 µg mg^−1^ at week 4, and then increases to 1.160 ± 0.033 µg mg^−1^ in week 10. Again, a similar decrease in sorption by week 4 and an increase in sorption by week 10 is observed for PET, PS, and HDPE.

The observed decrease in PAH concentrations from native nanoplastics to week 4 weathered nanoplastics that is followed by a slight increase by week 10 can be compared with the change in surface charge that is associated with the oxidation of the nanoplastics (Figure 1B,C). As the plastics become more negatively charged over time, their ability to sorb PAHs likely decreases initially due to an increased surface hydrophilicity of the nanoplastics. The subsequent increase in PAH loading with additional weathering from week 4 through week 10 is surprising. This increase in PAH loading may be due to an increase in porosity, and therefore surface area, as a result from fragmentation and restructuring of the nanoplastics surface as determined from BET isotherm data in our previous work.^[^ ^27^ ^]^ As the porosity and, correspondingly, the surface area increases, PAH sorption could become more favored. As will be discussed in greater detail later, the PAH loading measurements confirm that despite the oxidation of nanoplastics under simulated weathering conditions, the investigated nanoplastics retain their ability to enhance the effective aqueous concentrations of PAHs orders of magnitude beyond the carcinogens’ aqueous solubility limits.^[^ ^65^, ^66^, ^67^ ^]^ This finding is in good agreement with previous studies investigating PAH loading onto a blend of nanoplastics.^[^ ^27^ ^]^

### 2.3 Transport of Nanoplastics Across Intestinal Membranes and the Effects on PAH Bioavailability in an In Vitro Intestinal Membrane Model

We have previously shown that nanoplastics can increase the effective concentration of PAHs in aqueous media, which has immediate implications for PAH uptake and concentrations in the small intestine.^[^ ^27^ ^]^ In this study we set out to characterize how nanoplastics affect the transmembrane transport of PAHs in an in vitro intestinal membrane co‐culture model of Caco‐2 cancer epithelium cells differentiated by Raji‐B cells (M Cell model). In this co‐culture model, ≈70%–90% of the Caco‐2 cells typically develop distinct enterocyte‐like properties, while ≈10%–30% develop into M‐like cells induced by factors released from B cells.^[^ ^68^, ^69^ ^]^ We chose this M cell model over a conventional Caco‐2 intestinal membrane model, which has been widely used as model system for the study of intestinal interactions between nanomaterials, pharmaceuticals, xenobiotics, and free PAHs,^[^ ^70^, ^71^, ^72^, ^73^ ^]^ as the Caco‐2 model lacks the capacity to account for potential transepithelial transport of nanoplastics via M cells. As M cells are known to transport particles in the size range of the nanoplastics generated in this work,^[^ ^74^, ^75^, ^76^ ^]^ the M cell model provides a more realistic intestinal model for studying nanoplastics‐facilitated transepithelial PAH transport.

**Figure 3:**
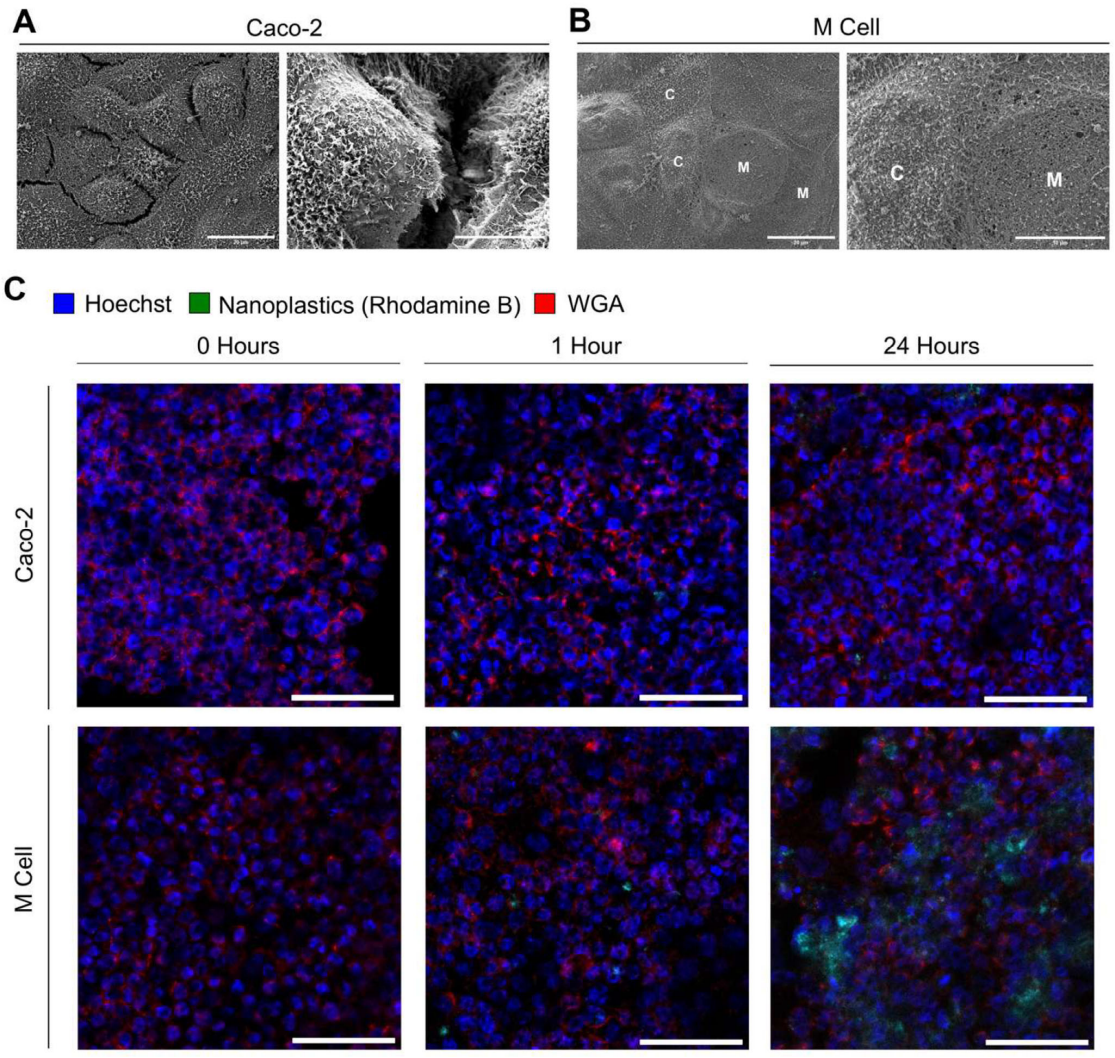
Characterization of intestinal membranes by SEM and confocal microscopy. A) SEM images of Caco‐2 only cell membrane at magnifications of 1.5k× (left) or 4k× (right). High magnification (right) reveals the surface microvilli. Scale bar: 20 µm (left) and 10 µm (right). B) SEM images of Caco‐2/Raji B co‐culture model (M Cell model) at 1.5k× (left) or 4k× (right) magnification. The area with a loss of microvilli indicates the presence of M‐cell (M), whereas microvilli‐rich areas indicate Caco‐2 cells (C). High‐magnification (right) images show smooth cell areas for the M‐cell phenotype. Scale bar: 20 µm (left) and 10 µm (right). C) Confocal images of the M cell model and Caco‐2 cells before addition of nanoplastics (0 h) as well as 1 and 24 h after the addition of nanoplastics. The cells were stained with Hoechst (blue), WGA (red), and nanoplastics stained with rhodamine B (green). Scale bar: 100 µm.

The cells were grown on a membrane in transwell inserts that separates an apical compartment (models the inside of the small intestine where nutrients are absorbed) from a basolateral compartment (models the blood stream and interior of the body) to investigate transport (Figure S3A, Supporting Information). We validated the successful formation of a cell monolayer on the transwell inserts for conditions that contain enterocytes (Caco‐2 only) as well as a mix of M‐like cells and enterocytes (M cell model) by scanning electron microscopy scanning electron microscopy (SEM) (**Figure** **3**A,B) and fluorescence microscopy (Figure 3C and Figure S3B, Supporting Information). Both techniques confirm the formation of continuous cell layers under both culturing conditions. The SEM images of the Caco‐2 only model show a continuous layer of cells, which all appear to contain a high percentage of microvilli characteristic of enterocytes (Figure 3A). Importantly, in the M cell model, we observe some cells with smooth surfaces (Figure 3B), characteristic of induced M cells, in addition to the Caco‐2 microvilli‐presenting enterocytes. These qualitative observations of the continuous monolayer were further validated with transepithelial electrical resistance (TEER) measurements of ≈150–250 Ω for the M cell model and ≈700 Ω for the Caco‐2 model, both of which are consistent with literature.^[^ ^21^, ^69^ ^]^

After validating the successful implementation of the intestinal membrane models, we characterized the concentration dependent effect of nanoplastics exposure (no PAH) on membrane integrity. To that end, we incubated the M cell membrane model with native PET nanoplastics concentrations ranging from 10 µg to 5 mg in 300 µL of cell culture medium added to the apical compartment (1.3 mL total volume in the transwell insert system) and measured TEER values (150–250 Ω) for up to 48 h (Figure S3C, Supporting Information). Only exposure to the highest nanoplastics input of 5 mg, which also increased the starting TEER values significantly, induced a significant drop in TEER indicative of a loss in intestinal membrane integrity over the course of the experiment. Unless otherwise noted, we use 500 µg of nanoplastics added to 300 µL of cell culture medium in the apical chamber (for effective PAH concentrations see Table S1, Supporting Information) of the transwell insert as this concentration was well below the threshold concentration for loss of membrane integrity and is a working concentration in good agreement with literature.^[^ ^14^, ^76^, ^77^ ^]^ In addition, although typical nanoplastics concentrations in water lie between 0.04 and 2 mg L^−1^,^[^ ^78^, ^79^, ^80^ ^]^ nanoplastics can enrich in the tissue of marine animals with some tissues containing concentrations of 0.1 mg g^−1^ or even ≈3 mg g^−1^.^[^ ^81^, ^82^ ^]^

Next, we tracked the uptake and distribution of nanoplastics in the intestinal membrane models through confocal fluorescence microscopy. To that end, 500 µg native PET nanoplastics were stained with rhodamine B, which has been used before as stain in micro‐ and nanoplastics studies.^[^ ^83^, ^84^, ^85^ ^]^ The nanoplastics were added to the apical chamber of the M cell model at 37 °C, the Caco‐2 only model at 37 °C (Figure 3C), as well as the M cell model at 4 °C (Figure S4A, Supporting Information), which should not support active transport at this reduced temperature.^[^ ^68^, ^74^ ^]^ Confocal images of cells were acquired after 0, 1, and 24 h of nanoplastics exposure (Figure 3C and Figure S4A,B, Supporting Information). After 24 h, the images reveal significantly higher concentrations of nanoplastics in the middle and bottom planes of the M cell model than for the Caco‐2 only model and the M cell model at 4 °C, suggesting that M cells enhance the uptake and transport of nanoplastics and that it is an active process since we see a clear difference between the M cell model at 37 and 4 °C. This observation was validated by examining images of the Caco‐2 only model, the M cell model, and the M cell model at 4 °C in the *x*‐, *y*‐, and *z*‐planes after 24 h (Figure S5, Supporting Information). These images show nanoplastics enrichment within some cells of the M cell model compared to the other models. Importantly, we also did not observe any change in TEER values over the course of 24 h.

**Figure 4:**
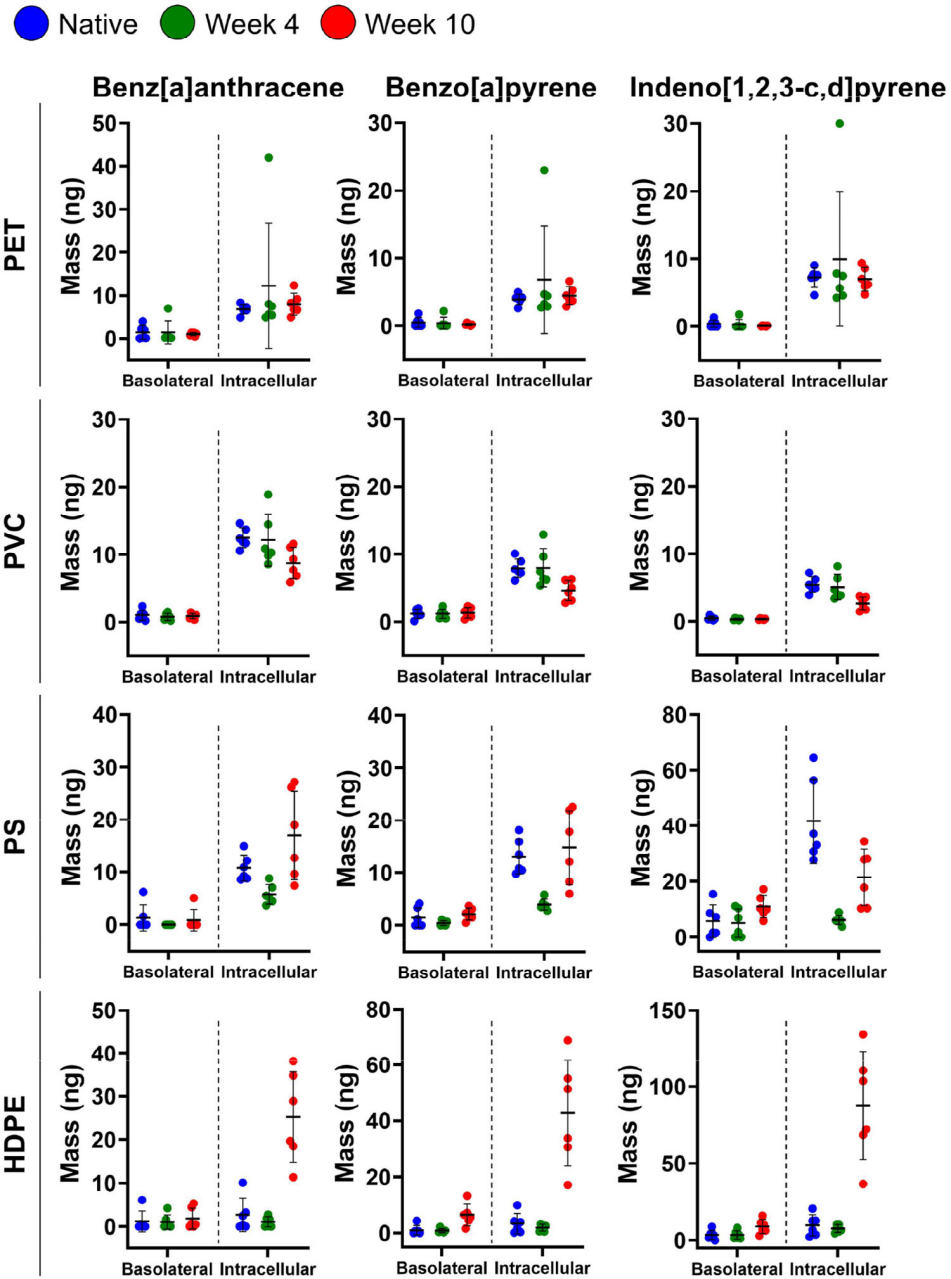
Quantification of nanoplastics‐mediated PAH uptake and transmembrane transport. Dot plots depict the mass (ng) of PAHs from PAH‐loaded nanoplastics that traversed across the M cell model cell membrane (Basolateral) or were taken up into cells (Intracellular) for native, and 4 and 10 weeks weathered PVC, PET, PS, HDPE nanoplastics. Error bars represent the mean mass ± SD (*n* = 6 biological replicates).

Nanoplastics‐mediated PAH transport was investigated in the M cell model using nanoplastics composed of PET, PVC, PS, or HDPE after exposure to UV weathering for 0 (native), 4, and 10 weeks (**Figure** **4**). We observed little to no change in TEER for the PAH‐loaded nanoplastics over an interval of 24 h (Figure S6, Supporting Information), nor a systematic change in Lactate Dehydrogenase (LDH) levels indicative of acute cell damage (Figure S7, Supporting Information).^[^ ^86^, ^87^ ^]^ However, despite an intact intestinal membrane, PAHs were detected intracellularly and in the basolateral compartment at the time of measurement (24 h after nanoplastics addition) (Figure 4). For native PAH‐loaded nanoplastics the intracellular PAH concentrations generally decrease in the order PS > PVC > PET > HDPE. The first three plastics are in order of decreasing hydrophobicity, but HDPE does not follow this trend. Our FT‐IR characterization suggested the presence of additives in this case, and the deviation of HDPE from the general trend of the intracellular PAH concentration may be due to the presence of these additives. Weathering of the nanoplastics was associated with measurable changes in the cellular and basolateral PAH concentrations. For PET, the intracellular PAH concentrations of nanoplastics weathered for 4 weeks are higher than for native nanoplastics. The intracellular PAH concentrations for PET weathered for 10 weeks appear higher than PET weathered for 4 weeks, but one outlier in PET weathered for 4 weeks drives the average higher. The PAH concentrations in the basolateral compartment are similar for all weathering conditions with a slight decrease observed for increasing weathering. In the case of PVC, native nanoplastics and nanoplastics exposed to 4 weeks of weathering achieve similar levels of cellular PAH concentrations, but the intracellular PAH concentrations decrease for PVC weathered for 10 weeks. The basolateral PAH concentrations for PVC remain at a similar level independent of the weathering condition. For PAH‐loaded PS nanoplastics, the intracellular PAH concentration drops for nanoplastics weathered for 4 weeks but then increases again for nanoplastics weathered for 10 weeks. The PAH concentrations in the basolateral compartment shows a similar trend. HDPE nanoplastics show a very strong increase in the intracellular PAH concentration for nanoplastics weathered for 10 weeks. Under these conditions, the PAH concentrations in the basolateral compartment are also noticeably increased. Even though HDPE nanoplastics weathered for 10 weeks do not exhibit remarkably higher PAH loadings (the week 10 PAH loadings are lower than for native HDPE), the interactions between nanoplastics and cells may in this case be affected by the ζ‐potential of the nanoplastics, which was more negative than for any other investigated condition, or compositional changes related to additives suggested by FTIR.

Overall, the PAH quantification shows that especially for HDPE, and to some degree for PS (week 10) and PET, photochemical weathering can enhance the capacity of nanoplastics to increase the intracellular PAH concentration, while weathering decreases uptake in the case of PVC. PAHs were consistently detected in the basolateral compartment, but the concentrations were lower than the intracellular levels. These results are comparable to previous studies of commercially available synthesized nanoparticles that reported low levels of transmembrane transport but significant intracellular uptake.^[^ ^14^, ^83^ ^]^ It is important to contextualize the weathering dependent uptake and transport data observed in this study with the physicochemical properties of the nanoplastics. The increasing surface charge (Figure 1B) of the nanoplastics due to weathering affect PAH sorption (Figure 2), but differences in the surface charge and surface chemistry will also affect the protein corona around the nanoplastics in biological media.^[^ ^88^ ^]^ This corona can influence nanoplastics interactions with and uptake into cells of the intestinal membrane.^[^ ^14^, ^88^, ^89^, ^90^ ^]^ Based on the data in Figures 2 and 4, we postulate that for some polymers a change in surface chemistry after UV weathering, as detected by a change in ζ‐potential (especially evident for HDPE), results in an increased intracellular uptake of PAH‐loaded nanoplastics. This model can account for increased intracellular PAH concentrations even if PAH loading decreases as a function of weathering.

Even though the trends between weathering and the cellular and/or basolateral PAH concentrations vary depending on the plastics material, it is important to note that all PAH‐loaded nanoplastics achieve intracellular and basolateral PAH concentrations that are much higher than those obtained with free PAHs (Water_PAH_) added to the apical compartment of the transwell insert, in the same manner as PAH‐loaded nanoplastics, at the respective saturation limits. For Water_PAH_, both cellular and basolateral PAH concentrations remain below the detection limit. In addition, intracellular PAH levels are significantly higher for PS nanoplastics in the M cell model compared to the Caco‐2 only membrane or the M cell model at 4 °C control (Figure 4 and Figure S8, Supporting Information), indicative of active uptake by M cells. Consequently, we attribute the gain in intracellular PAH concentration to the uptake of PAH‐loaded nanoplastics by M cells. Only indeno[1,2,3‐c,d]pyrene shows higher basolateral concentrations in the M cell model at 4 °C than in the Caco‐2 only model at 37 °C (Figure S8, Supporting Information). The lack of a systematic increase for all PAHs indicates that active transport via M cells also dominates the PAH concentration increase in the basolateral compartment for the data shown in Figure 4.

### 2.4 Characterizing the Transcriptional Response of the Intestinal M Cell Model to PAH‐Loaded Nanoplastics

**Figure 5:**
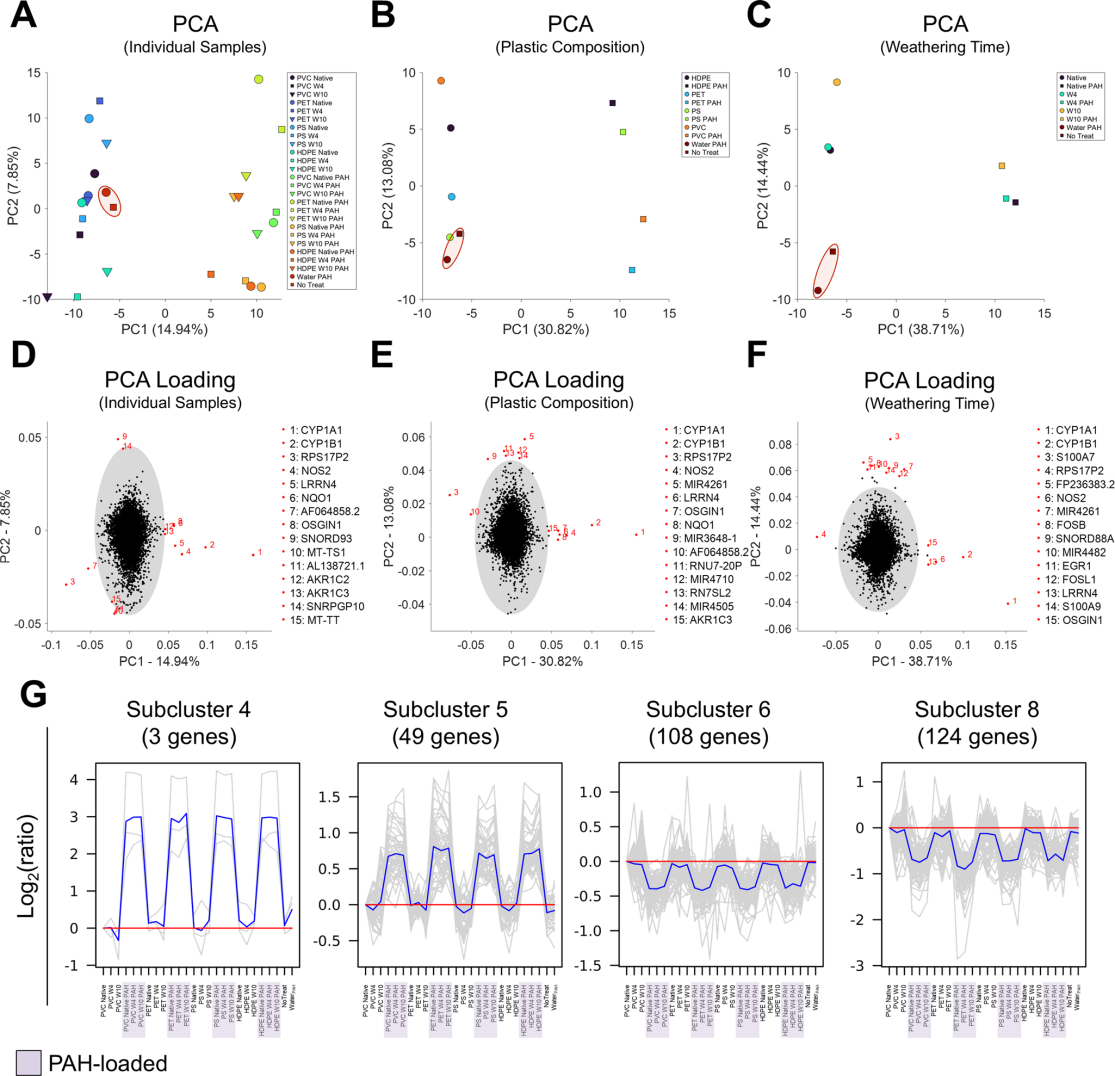
PCA of all conditions and subclustering of differentially expressed genes (DEGs). A–C) 2D PCA plot (PC2 vs PC1) for A) all individual samples as well as Water_PAH_ and NoTreat controls, B) samples grouped by composition HDPE, PVC, PS, PET with and without PAH loading as well as Water_PAH_ and NoTreat controls, C) samples grouped by 0 weeks weathering (native), 4 weeks weathering (W4), and 10 weeks weathering (W10) with and without PAH loading as well as Water_PAH_ and NoTreat controls. Water_PAH_ and NoTreat controls are circled in red. D–F) PC2 versus PC1 Loading scores plotted of 2D PCA plots generated in (A)–(C). Gray shaded area denotes 10 SD from the origin. The top 15 genes furthest away from the origin are highlighted and numerically labeled in red. G) Subclusters of genes captured from hierarchical clustering heatmap in Figure S11 (Supporting Information). Gray lines represents gene expression relative to the corrected expression levels for the individual genes and blue line represents the mean for all genes in the cluster.

The lack of systematic changes in TEER or LDH values for the intestinal membrane models under nanoplastics exposure does not preclude the existence of more subtle cellular responses to PAHs and/or nanoplastics on the transcriptional level. Since we were able to observe indications of slightly increased reactive oxygen species (ROS) levels in confocal images of cells exposed to nanoplastics‐loaded with and without PAHs (Figure S9, Supporting Information), we performed bulk RNA sequencing to illuminate the transcriptional response of the M cell model to nanoplastics, Water_PAHs_, and PAH‐loaded nanoplastics exposure. Membranes were exposed to PET, PVC, HDPE, or PS with no weathering (native), 4 weeks or 10 weeks of weathering. All nanoplastics conditions were considered with and without PAH loading and for an exposure duration of 24 h. We also included cells exposed to free PAHs at their respective solubility limit in water (Water_PAH_, 3 biological replicates) and untreated membranes as no treatment controls (NoTreat, 4 biological replicates). Pearson Correlation plots of gene expression for the different conditions were generated to assess the similarity between the samples (Figure S10A–C, Supporting Information). Pairwise correlations between individual test samples, samples grouped by plastics, and samples grouped by weathering time revealed that within the PAH‐loaded nanoplastics group, samples were more correlated to each other than to their unloaded starting state, indicating a strong effect of PAHs on the transcriptional response. Since our analysis involved >50000 different genes, we reduced the dimensionality of the dataset by principal component (PC) analysis (PCA) to alleviate the visualization of the data and to identify systematic differences between the selected conditions. The PC1‐PC2 plot for all conditions in (**Figure** **5**A) shows a clear separation of PAH‐loaded and unloaded nanoplastics along PC1, with Water_PAH_ and NoTreat controls clustering with the unloaded nanoplastics. PC2 achieves some separation based on type of plastics and weathering time. PCA performed on samples grouped by composition of plastics (no distinction based on weathering time) (Figure 5B) enhances the separation along PC1 and PC2. Lastly, PCA performed on samples grouped by weathering time (no distinction based on composition) (Figure 5C) shows little separation between native nanoplastics and samples weathered for 4 weeks, but good separation between these two groups and samples weathered for 10 weeks along PC1 and PC2 for both PAH‐loaded and unloaded samples. The PCA analysis confirms systematic differences in the gene expression response to PAH‐loaded/unloaded nanoplastics (separation along PC1 in Figure 5A–C) and to a lesser degree between nanoplastics of different compositions and different degrees of weathering (separation along PC2 in Figure 5A–C).

The loading scores of PC1 and PC2 (Figure 5D–F) contain information about the genes that contribute most strongly to the separation of the data along those principal components. The function of each gene was determined using the NCBI Gene database, MyGene.info database, and the GeneCards database. In PC1 CYP1A1 and CYP1B1, which play a role in detoxification processes and are both involved in the metabolic degradation of PAHs and xenobiotics,^[^ ^91^, ^92^ ^]^ have high positive scores, indicative of upregulation of the related pathways. Genes like NOS2 and NQO1, which are involved in inflammatory immune responses to injury and oxidative stress, are also remarkable and have above‐average positive loading scores, indicative of inflammatory or stress‐related pathways being activated. The loading scores show that differences along PC1 in response to PAH‐loaded nanoplastics have strong contributions from variability related to detoxification and stress. For PC2, which seems to separate samples based on weathering duration or chemical composition, SNORD93 and SNRPGP10 have high positive loading scores. These genes are involved in RNA processing and regulation. MT‐TS1 and MT‐TT, both mitochondrial tRNA genes, have negative loading scores, which suggests a decrease in mitochondrial activity or shifts in energy metabolism in cells treated with nanoplastics of varying compositions or weathering durations. Overall, variations in RNA processing and mitochondrial function likely contribute most to the separation along PC2. The fact that a few genes stand out and exhibit particularly eminent roles in PC1 and PC2 also aligns with the profiles of generated violin plots, box plots, and fragments per kilobase of transcript per million mapped reads (fpkm) density distribution plots, which all independently show a relatively small percentage of genes driving the majority of the cellular response to PAH exposure (Figure S10D–F, Supporting Information).

Differential gene expression analysis, relative to NoTreat cells, was performed to further quantify trends in the transcriptional response to PAHs and nanoplastics. A total of 712 unique genes were identified as differentially expressed (DEGs, |Log_2_(Fold Change)| ≥ 1 and adjusted *p*‐values (*p*_adj_) ≤ 0.05). To investigate relationships between samples and gene expression, a hierarchical clustering heatmap of fpkm values was generated detailing clustering of both genes and samples (Figure S11A, Supporting Information). Hierarchical clustering dendrograms were generated for individual samples and also for grouped samples, including plastic composition or weathering time. These plots demonstrate clear separation of nanoplastics based on PAH loading, composition and weathering time (Figure S11B–D, Supporting Information). Interestingly, the similarity of NoTreat controls to other samples decreases in the order Water_PAH_ controls > unloaded nanoplastics > PAH‐loaded nanoplastics (Figure S11B, Supporting Information). As was observed in PCA, native nanoplastics and nanoplastics weathered for 4 weeks show overall greater similarity than nanoplastics weathered for 10 weeks (Figure S11B,D, Supporting Information). The PAH‐loaded nanoplastics samples differ from all other nanoplastics samples (Figure S11B–D, Supporting Information). As in the case of the unloaded nanoplastics, PAH‐loaded nanoplastics with no or 4 weeks of weathering are more similar to each other than to PAH‐loaded nanoplastics weathered for 10 weeks (Figure S11B,D, Supporting Information). Interestingly, these data also suggest that the similarity in the gene expression between unloaded nanoplastics decreases in the order PS < HDPE < PET ∼ PVC and for PAH‐loaded nanoplastic in the order HDPE ∼PS > PVC > PET (Figure S11C, Supporting Information). For PAH‐loaded nanoplastics, the similarity quantified as Euclidean Distance follows the intracellular PAH uptake (Figure 4). The hierarchical clustering confirms the general trends from the PCA analysis that PAH loading, type of nanoplastics, and weathering time all impact the transcriptional response, but that the PAH loading has a particularly significant impact.

**Figure 6:**
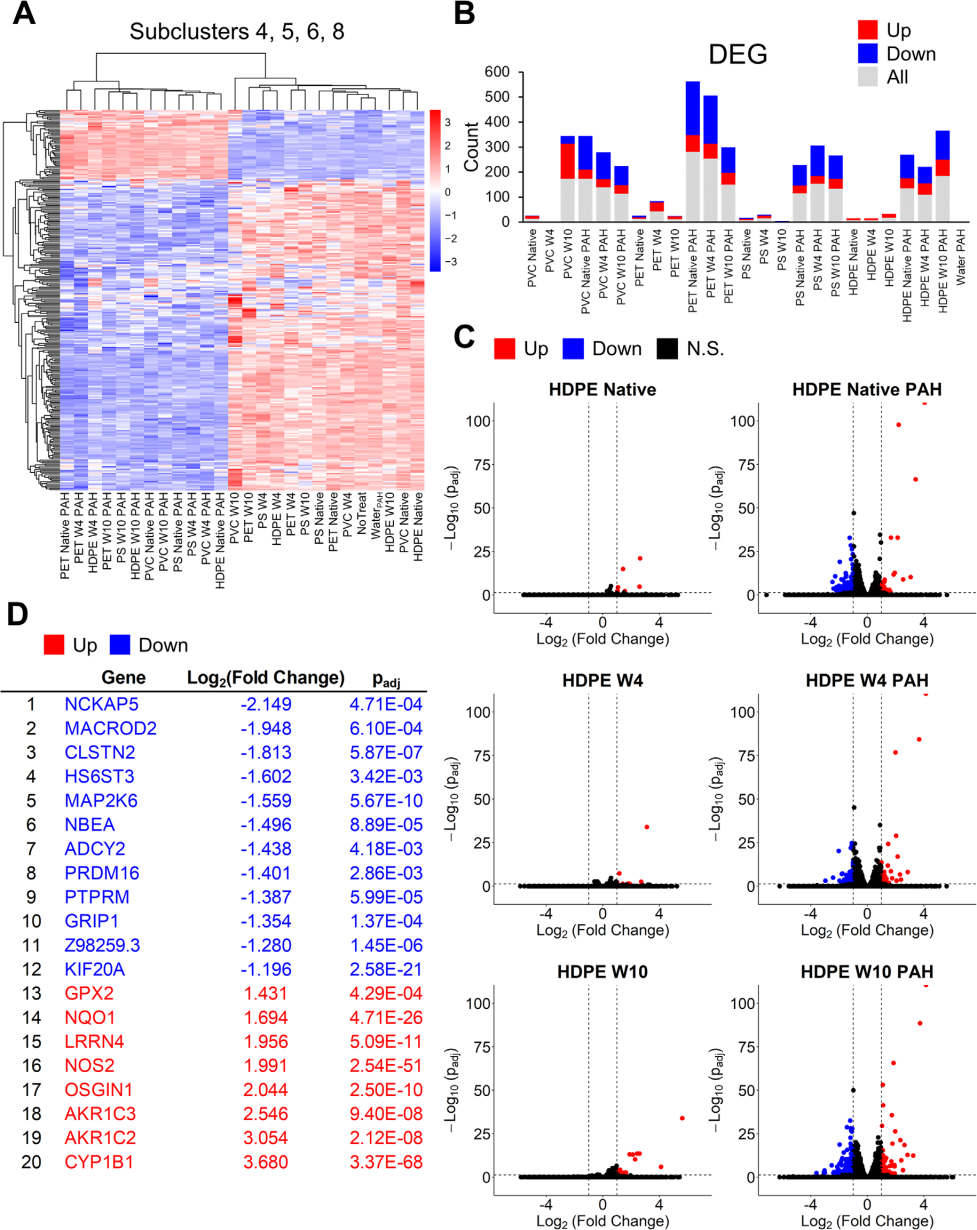
DEG visualizations of transcriptomic changes in treated membranes. A) Two‐way hierarchical clustering heat map of RNA‐Seq transcriptome analysis of the genes from subclusters 4, 5, 6, 8 (in Figure 5G). *x*‐axis represents conditions, where *y*‐axis represents genes. Data Log_2_ normalized. Scale bar illustrates the intensity of Log_2_‐transformed expression value. B) Bar chart of the DEG count for upregulated (Up), downregulated (Down), and sum (All) of all genes relative to the NoTreat control. |Log_2_(Fold Change)| ≥ 1 and *p*_adj_ ≤ 0.05. C) Representative volcano plots displaying DEGs for native, week 4 (W4), week 10 (W10) HDPE with and without PAHs relative to the NoTreat control group. |Log_2_(Fold Change)| ≥ 1 and *p*_adj_ ≤ 0.05. Red dots represent genes above this threshold in the positive direction (Up), blue dots above this threshold in the negative direction (Down), black dots do not meet the criteria (N.S.). Remaining plots for plastics are provided in Figure S12 (Supporting Information). D) List all DEGs that are shared across all nanoplastic samples loaded with PAHs (12), but not observed in any unloaded nanoplastic samples (12). |Log_2_(Fold Change)| ≥ 1 and *p*_adj_ ≤ 0.05. Reporting average Log_2_(Fold Change) and *p*_adj_ values.

The hierarchical clustering heatmap can provide more granular information if it is broken up into subclustering plots containing genes with similar trends in expression levels under different conditions (Figure 5G). In these graphs, centered Log_2_(ratio) values were plotted as a function of each condition and were connected by straight lines. A blue line is included for the mean value of all genes in the cluster relative to the corrected expression level under different experimental conditions. Gray lines represent gene expression relative to the corrected expression levels under different experimental conditions. Subclusters generated by h‐clustering broke up the genes into eight subclusters. Subclusters 4 (3 genes), 5 (49 genes), 6 (108 genes), and 8 (124 genes) achieve a clear separation and grouping of samples: PAH‐loaded nanoplastics separated from unloaded samples with Water_PAH_ and NoTreat controls. Depicted as a condensed hierarchical clustering heatmap (subclusters 4,5,6,8), subclusters 4 and 5 appear to have upregulated genes in PAH‐loaded samples and subclusters 6 and 8 appear to have downregulated genes in PAH‐loaded samples (**Figure** **6**A). The gene expression analysis suggests that cells exposed to PAH‐loaded nanoplastics are experiencing significant stress, as reflected by the upregulation and downregulation patterns observed across the subclusters. Subclusters 4 and 5, which contain upregulated genes, indicate a robust cellular response to oxidative stress and toxic stimuli. In subcluster 5, many of the genes are involved in oxidative stress response (TXNRD1, NQO1, GSR), detoxification pathways (AKR1C3, ABCG2), and antioxidant defense (GPX2, HMOX1, GCLM). The upregulation of these genes suggests that the cells are encountering increased oxidative stress due to the presence of carcinogenic PAHs and are activating protective mechanisms to counteract potential damage. This response is further validated by subcluster 4, where the upregulation of genes CYP1A1, CYP1B1, and NOS2 signals increased metabolism and associated oxidative stress. In addition to the oxidative stress response, subcluster 5 contains genes related to cell growth, apoptosis, and inflammation (MAP3K5, BMP6, CEBPD), suggesting a broader cellular response to environmental stressors such as toxins, infections, or injury. The activation of these pathways indicates that the cells are not only trying to detoxify the harmful PAHs but are also managing potential disruptions in growth and inflammatory processes. In contrast, subclusters 6 and 8 downregulated genes in response to PAH‐loaded nanoplastics. Subcluster 6 shows the downregulation of genes involved in cellular growth and immune responses (PLK1, KIF14, PTPRM, TGM2), suggesting that these important pathways are being suppressed. Subcluster 8 features downregulation of genes related to mitochondrial function and energy metabolism (XDH, TPK1). This may indicate that the cells are deprioritizing growth and immune functions as they focus on managing nanoplastics and especially PAH‐induced stress.

Next, expression levels of all 712 unique differentially expressed genes (relative to NoTreat controls) were plotted as a bar graph (Figure 6B) and individual volcano plots (Figure 6C and Figure S12, Supporting Information) to determine trends in overall expression. The most significant changes in gene expression are observed for PAH‐loaded nanoplastics, with both significant up‐ and downregulation of genes. Importantly, no gene shows a statistically significant upregulation or downregulation of genes in the Water_PAH_ control relative to the NoTreat samples, which suggests that aqueous solutions saturated with PAHs do not reach the carcinogen concentration required to illicit a detectable cellular response under the chosen experimental conditions.

Of the 712 unique genes that show differential expression, no gene appears to be up‐ or downregulated across all unloaded nanoplastics samples. However, a number of genes shows significant upregulation in >50% of unloaded nanoplastics samples. Upregulated genes observed were involved in differentiation and proliferation in the context of epithelial remodeling (EMP1), endothelial cell adhesion (CDH5), transcription factors involved in differentiation and survival in response to external stimuli (EGR1, FOSB), and inflammatory response and immune system regulation cytokines (IL32). Collectively, these genes suggest cells are initiating a cellular response involving stress‐related signaling and inflammation in response to nanoplastics. In addition, CDH5 expression implies potential barrier function loss, while EMP1 could be a compensatory cellular response to help improve and maintain barrier function. For PAH‐loaded nanoplastics, 20 genes show changes across all of the PAH‐loaded nanoplastics samples with no significant changes in unloaded samples (Figure 6D). In addition to the genes already discussed above, downregulated genes in response to PAH‐loaded nanoplastics include NCKAP5, MACROD2, MAP2K6, KIF20A, CLSTN2, ADCY2, PTPRM. These genes are associated with a range of diverse functions. NCKAP5 and MACROD2 are involved in actin cytoskeleton organization and DNA repair. MAP2K6 is related to stress‐activated MAP kinase signaling, KIF20A to mitotic spindle organization and cytokinesis during cell division, and CLSTN2 to Ca^2+^ binding regulation. ADCY2 is involved in cAMP signaling, and PTPRM in cell adhesion. Upregulated genes include AKR1C3, AKR1C2, and NQO1, which play roles in detoxifying aldehydes, steroids, and xenobiotics, as well as GPX2, an enzyme that protects cells from oxidative damage, and OSGIN1, an oxidative stress‐induced growth inhibitor. Overall, the breadth of differential gene expression induced by the delivered PAHs not only confirms an increase in oxidative stress, inflammation, and PAH metabolism responses (GPX2, OSGIN1 and CYP1A1, CYP1B1, NOS2 discussed above and in previous sections), but also suggests a disruption (decrease) in basic cellular functions related to cell division, signaling, cellular coordination, and structure.

Systematic changes as a function of weathering without PAH loading, comparing the average Log_2_(Fold Change) expression of each plastic (HDPE, PET, PS, and PVC) for native and W10 plastics independently, are also observed. W10 shows a more pronounced immune response, with higher expression of immune‐related genes (S100A7, C3, IL6, IL32). Cellular stress markers (JUN, ATF3, NR4A1, FOS family genes) are also more upregulated in W10, indicating increased stress adaptation. Structural proteins (KRT17, ITGA2, THBS1) are upregulated in W10, suggesting cytoskeletal remodeling. However, mitochondrial function (MT‐ATP8, MT‐TL1, MT‐ND1, MT‐TH) and energy metabolism appear to be more downregulated in native nanoplastics compared to W10 nanoplastics.

With the differential expression analysis data, we investigated Kyoto Encyclopedia of Genes and Genomes (KEGG), Gene Ontology (GO), and Disease Ontology (DO) databases. Investigation of KEGG and GO databases revealed a number of relevant biological pathways, while DO did not provide noteworthy assignments for this cell culture model (Figures S13 and S14, Supporting Information). Nanoplastics were grouped together as native, weathering for 4 weeks, and weathering for 10 weeks with or without PAH loading. We also investigated Water_PAH_ controls, cells exposed PAHs at the solubility limit in aqueous medium. Each sample was relative to NoTreat cells. Many pathways were flagged as statistically significant. However, the data highlight the importance of avoiding over‐reliance on pathways that may not be biologically relevant to the M cell model. For example, KEGG analysis identified ovarian steroidogenesis pathways for PAH‐loaded nanoplastics, which are typically associated with reproductive tissues, as well as osteoclast differentiation. However, the appearance on this list may suggest commonalities in the underlying cellular response. For example, GO identified steroid metabolic processes. This makes sense as Cytochrome P450 (CYP450) enzymes assist in the metabolization of steroids, drugs, toxins, and other compounds such as PAHs. Consistent with the literature,^[^ ^93^ ^]^ KEGG identified immune responses, such as IL‐17 signaling, for both loaded and unloaded nanoplastics. The IL‐17 signaling pathway is associated with immune responses and inflammation, which could be triggered by cellular stress or environmental exposure. In addition, PAH‐loaded nanoplastics exhibited pathways related to metabolism and detoxification, such as metabolism of xenobiotics by cytochrome P450, steroid hormone biosynthesis, and chemical carcinogenesis. However, since this study investigates cellular responses on a short time scale (less than 24 h), it is unclear how much one should read into the identification of carcinogenic processes. GO was in good agreement with these assignments. However, in addition, GO suggested potential changes in cellular structure and transcription activity post‐exposure to nanoplastics. Collectively, these data highlight that in all groups, inflammation‐related pathways were involved as part of the response to nanoplastics, while detoxification pathways were involved in the management of PAHs. Importantly, this analysis did not identify any pathways for membranes exposed to free PAHs (Water_PAHs_) at their respective saturation limits in aqueous solution, further highlighting the potential of nanoplastics to enhance PAH‐related responses by increasing the effective PAH concentration.

## 3 Conclusion

Plastic waste in the ocean is a growing environmental concern, and the degradation of plastics into micro‐ and nanoplastics risks increased exposure of marine organisms and poses a potential threat to the food chain. In this work we have investigated the complex interplay between nanoplastics, photochemical weathering, PAHs sorption, and its effect on the transcriptome of a nanoplastics‐treated intestinal membrane model. Even after prolonged photochemical weathering associated with chemical changes indicative of oxidation, nanoplastics were observed to achieve significant loading with PAHs. Our studies revealed differences in PAH uptake for nanoplastics of different polymeric composition and weathering state. But despite some differences in the physicochemical evolution of the different nanoplastics under weathering conditions, all nanoplastics samples facilitated much higher PAH uptake and transmembrane transport than was possible for free PAHs in water (Water_PAHs_), underlining the potential of nanoplastics to mobilize PAHs. Transcriptional profiling of the response of a M cell model provided evidence of PAH‐loaded nanoplastics induced cell dysfunction driven by the need to manage oxidative stress and detoxification at the cost of disrupting mitochondrial function and energy metabolism. While PAH‐loaded nanoplastics had obvious effects at the transcriptional level, even more than half of unloaded nanoplastics (no PAH loading) conditions were also found to induce changes in gene expression related to signaling, inflammation, and structural remodeling as result of stress from nanoplastics exposure. Given the widespread and pervasive nature of nanoplastics in marine environments, these observations are noteworthy and corroborate concerns about the environmental risks posed by nanoplastics alone and especially in combination with hydrophobic environmental toxins, such as PAHs. At the same time, a detailed understanding of the interactions between environmental toxins and nanoplastics provides opportunities for identifying materials properties that could be used to minimize their effect.

This work motivates future studies that validate the observed gene expression changes in response to nanoplastics, with and without PAH loading, through techniques such as ELISA, which was previously applied to characterize the response of intestinal membranes to micro‐ and nanoplastics,^[^ ^77^, ^94^ ^]^ qPCR, and Western blotting to confirm the biological relevance of the identified transcriptional changes. Furthermore, more advanced in vitro models, such as those that include mucus generation in the small intestine, as well as functional in vivo assays, including gene knockdown or overexpression studies, are needed to provide further insight into the roles of other environmental factors of the intestine and of specific genes involved in the response to nanoplastics. Many questions remain open, and this work advocates for more research into strategies to mitigate the impact of nanoplastics pollution and improve the protection of both marine ecosystems and human health.

## 4 Experimental Section

### Nanoplastics Synthesis and Weathering

Plastics utilized for this study were from a milk jug (HDPE), a single‐use Poland Spring water bottle (PET), a red Solo Cup (PS), and a white pipe (PVC). Nanoplastics were generated as in a previous study,^[^ ^27^ ^]^ but for one plastic sample at a time. Briefly, a household blender (Hamilton Beach) broke the plastic samples into small debris. The debris was suspended in 20 mL of deionized (DI) water containing 15 cm^3^ worth of 0.3 mm diameter YSZ balls (Inframat, catalog no. 4039GM‐S003). This cocktail was placed in a Semi‐Clear HDPE Plastic Vial (McMaster‐Carr, catalog no. 4280T31) and milled using standard workshop ball mill for intervals of 15 min on, 5 min off. During the off period, an industrial fan was used to cool the mixture and mill itself to ensure these did not induce thermal degradation or weathering. The resulting mixture was filtered through an Advantech L3‐S100 Standard Sieve, Stainless Steel Mesh, 0.150 mm sieve (Advantech, catalog no. EW‐59948‐41), to remove the YSZ balls. To remove the aqueous solution from the nanoplastics, plastics were collected using a 100 nm filter (Thermo Fisher Scientific, catalog no. 568‐0010) and then washed by centrifugation. Plastics were re‐suspended in 3.5 L ocean water (Coast Guard Beach, Massachusetts, 41.8435° N, 69.9482° W), which was first filtered with a 100 nm filter to remove debris from the ocean. Nanoplastics were weathered by ultraviolet (UV) radiation using a 160 Watt Lucky Herp Reptile UVA/UVB Mercury Vapor Bulb (Lucky Herp, catalog no. R115) emitting 1800 µW cm^−2^ UVB, reported by the manufacturer, from a distance of 30 cm on center of the beaker. Samples were exposed at intervals of 4 h “on” and 6 h “off,” to keep the water temperature under 45 °C so as to not induce thermal degradation or weathering. During “on” cycles, samples were also stirred. Evaporated water was replaced with deionized water periodically to ensure salt content remained stable. Samples were collected at weeks 0 (native), 1, 2, 3, 4, 5, 8, and 10 using a fresh 100 nm filter, as above, and then resuspended in DI water until use for future analysis.

### DLS Measurements for Hydrodynamic Diameter and ζ‐Potential

Size and ζ‐potential measurements were determined by suspending 0.5 mg mL^−1^ and 1 mg mL^−1^, respectively, nanoplastics sample in DI water and measuring on a Zetasizer Nano ZS90 (Malvern, Worcestershire, UK).

### Fourier‐Transform Infrared Spectroscopy

Between 2 and 5 mg of nanoplastics were dried under vacuum on a glass slide overnight. Nanoplastics were scraped off the slide using a razor blade and pressed into a pellet using the built‐in adaptor of the Nicolet Nexus 670 FTIR with attenuated total reflection (ATR), which was utilized to collect the data. Parameters: KBr detector, KBr beam splitter, Range 4000 to 400, optical velocity 0.6329, Aperture 34.

### X‐Ray Photoelectron Spectroscopy

Nanoplastics were drop‐coated onto a silicon wafer (University Wafer, catalog no. 809) and dried under vacuum for 24 h. Measurements were collected on a Thermo Scientific K‐Alpha+ instrument and corrected using “Smart” background.

### PAH Loading and Quantification

Nanoplastics were suspended in 1 mg mL^−1^ of DI water:methanol mix (40:60) containing benz[a]anthracene (Sigma‐Aldrich, catalog no. 48563) [20 µg mL^−1^], benzo[a]pyrene (Sigma‐Aldrich, catalog no. 51968‐50MG) [20 µg mL^−1^], and indeno[1,2,3‐cd]pyrene (Sigma‐Aldrich, catalog no. 94377‐10MG) [20 µg mL^−1^] and shaken in the dark for 3 d. Solutions were spun down, the supernatant was removed, and then the pellet was washed three times with DI water at 4000 *g* for 10 min. The PAH‐loaded plastics were then dried under vacuum. The plastics were resuspended in 250 µL of hexane, transferred to vials (Agilent, catalog no. 5188‐6592, 5182‐0724), and analyzed using gas chromatography‐mass spectrometry (Agilent 6890N or 7890B, Agilent 7683B Sampler), 10.0 µL syringe (Agilent catalog no. 5181‐3321), and Agilent J&W DB‐5 ms Ultra Inert 30 m × 0.25 mm, 0.25 µm column (catalog no. Agilent p/n 122‐5532UI) or a Zebron ZB‐5MSplus 30 m × 0.25 mm, 0.25 µm column (catalog no. 7HG‐G030‐11). Parameters included: 2.0 µL injection, *Carrier*: Helium 45 cm s^−1^, constant flow, *Inlet*: Pulsed splitless; 40 psi until 0.7 min, purge flow 30 mL min^−1^ at 1.25 min, *Oven*: 60 °C (1 min) to 320 °C (25 °C min^−1^), hold 3 min, *Detection*: MSD source at 250 °C, quadrupole at 180 °C, transfer line at 280 °C. SIM mode benz[a]anthracene (224, 225, 226, 227, 228, 229), benzo[a]pyrene (248, 250, 251, 252, 253) and indeno[1,2,3‐cd]pyrene resolved at (272, 274, 275, 276, 277). Concentrations were calculated by fitting the area under the curve for each peak to a standard curve generated for concentrations ranging from 50 ng mL^−1^ to 5 µg mL^−1^. Data were plotted in GraphPad Prism.

### Cell Culture

The human colon carcinoma Caco‐2 cell line (American Type Culture Collection, HTB‐37, derived from a 72‐year‐old, white, male with colorectal adenocarcinoma, Passages 0–16) was maintained in EMEM + l‐glutamine (American Type Culture Collection, catalog no. 30‐2003) containing 20% fetal bovine serum (FBS, American Type Culture Collection, catalog no. 30‐2020) and 1% penicillin‐streptomycin solution (pen/strep, American Type Culture Collection, catalog no. 30‐2300). The Raji B cell line (American Type Culture Collection, CCL‐86, derived from 11‐year‐old, black, male, with Burkitt's lymphoma, Passages 0–20) was maintained in RPMI1640 + l‐glutamine (Gibco, catalog no. 11875085) containing 10% FBS and 1% pen/strep. The Caco‐2 cells were seeded at a density of 1×10^5^ cells per well onto a transwell membrane cell culture insert (Corning, catalog no. 3415, 0.33 cm^2^ surface area, 3 µm pore size, ≈75–85 Ω in cell culture medium at 37 °C).^[^ ^69^ ^]^ Media was changed every 2–3 d through day 10–12, determined by the point at which cells achieved a density of 700 Ω based on the literature.^[^ ^21^, ^69^ ^]^ At this point, 1 × 10^5^ Raji B cells were introduced into the basolateral compartment of the transwell insert. Media was gently changed every 3 d until day 18–21, as appropriate, so that average TEER measurements ranged between 150 and 250 Ω, which is consistent with M cell development.^[^ ^69^ ^]^ Inserts were then washed and resuspended with phenol free RPMI1640 media containing 10% FBS and 1% pen/strep in a new 24‐well plate to be used for experiments. Both cell lines were negative for mycoplasma (Invivogen, catalog no. REP‐MYS‐10) after completion of all experiments reported in this study.

### TEER Measurements

TEER measurements were performed using a Millicell‐ERS Voltameter (Millipore Sigma, catalog no. MERS 00001 and MERSSTX01). On the day of the experiment, electrodes were sterilized using 70% ethanol and were washed with sterile cell culture media prior to taking a measurement in an insert. Measurements were taken immediately after plates were removed from the 37 °C incubator. At each time point, three measurements were taken per sample and then averaged. TEER values were monitored over 24 h. The percent change in TEER values at each time point was calculated relative to the initial starting measurement (*t* = 0 h). Data plotted in GraphPad Prism.

### LDH Measurements

LDH was quantified using a CyQUANT LDH Cytotoxicity Assay kit (Invitrogen, catalog no. C20300) according to vendor instructions with some modifications. Briefly, 50 µL of cell growth medium was collected from the apical compartment of the transwell inserts and placed in a 96‐well plate. Then, 50 µL of LDH reaction mixture was added and incubated for 30 min at room temperature in the dark. Finally, 50 µL stop solution was added. Within 1 h, the absorbance was measured at 490 and 680 nm on a plate reader (Molecular Devices, model no. SpectraMax M5). Measurements at 490 nm were corrected by subtraction of 680 nm readings. The percentage of LDH activity change was normalized by the no‐treatment control group. Data plotted in GraphPad Prism.

### Scanning Electron Microscopy

SEM was used to analyze the surface morphology of both the Caco‐2 and M Cell models. Prior to imaging, the samples were fixed using 37% formaldehyde in ethanol for 2 h. Then the samples were dehydrated using graded ethanol solutions of 80%, 90%, and 100% for 20 min each. After this, the samples were further dehydrated using hexamethyldisilazane for 30 min two times. The samples were kept in the desiccator for 2 h before they were cut carefully and transferred to silicon wafers. The samples were sputter coated with a thin gold Au/Pd film (Cressington Scientific Instruments, model no. Cressington 108). SEM images were acquired on a Zeiss Gemini 360 at 3 kV. To generate representative nanoplastic images, nanoplastics suspended in DI water were drop coated onto silicon wafers and dried under vacuum. Samples were sputter coated as above and imaged using a Thermo Scientific Phenom XL G2 Desktop SEM at 10kV.

### Confocal Microscopy

For experiments requiring Rhodamine B‐loaded nanoplastics, Rhodamine B dissolved in DI water at a concentration of 20 µg mL^−1^. The solution was added to the nanoplastics (500 µg mL^−1^ of plastic) and placed on a shaker overnight in the dark. Nanoplastics were washed with sterile cell culture media three times at 4000 *g* for 10 min. 500 µg of nanoplastics were then added to the apical compartment of the transwell insert. At select intervals, samples for confocal microscopy were washed twice with DPBS, before the nucleus was stained with Hoechst 33342 (Thermo Scientific, catalog no. 62249) following the vendor's protocol. After the samples were washed twice with DPBS, WGA was stained with Alexa Fluor 488 (Invitrogen, catalog no. W11261) following the vendor's protocol. For ROS measurements, the samples were washed and stained with ROX (Invitrogen, catalog no. C10448) following the vendor's protocol. Samples were fixed by incubating in 4% formaldehyde solution for 15 min. The samples were washed and imaged. The confocal images were taken using Olympus Model FV3000 scanning confocal microscope with an air objective of 20×. The images were processed using ImageJ. Nonlinear signal modifications were performed to individual channels before merging.

### PAH‐Loaded Nanoparticle Transport Quantification

At 24 h after the addition of PAH‐loaded nanoplastics, media from the apical chamber of the transwell insert was harvested and placed in a glass scintillation vial. The cell‐containing membrane was washed, cut out of the insert, and placed in a GC‐MS vial. The basolateral compartment solution was removed and also placed in a glass scintillation vial. Cell culture media from the apical and basolateral compartments were evaporated under vacuum. Then, the apical compartment‐containing vial was resuspended in 500 µL of hexane, and the basolateral compartment‐containing vial was resuspended in 250 µL of hexane. The volumes were then transferred to GC‐MS vials. The cell membrane‐containing GC‐MS vial was then resuspended in 500 µL hexane. Samples were analyzed by GC‐MS as above, and the concentration of PAH in each compartment was determined by fitting to a standard curve. Intracellular and basolateral concentrations were baseline corrected by NoTreat cell membranes. Data plotted in GraphPad Prism.

### RNA Sequencing

RNA was extracted from cells using the RNeasy Micro Kit (Qiagen, catalog no. 74004) using manufacturer instructions with slight modification. Briefly, cell culture media was removed from the basolateral and apical chambers. Transwell inserts were washed with cell culture media two times to remove nanoplastics and free PAHs (Water_PAHs_). Then, 200 µL buffer RLT was added to the insert, cells were scraped, and then transferred to a RNeasy MinElute spin column. This was repeated with an additional 150 µL Buffer RLT (350 µL total). 350 µL of 70% ethanol was added to the RNeasy MinElute spin column and the entire 700 µL volume was mixed by pipetting. The remaining steps were performed as described by the manufacturer. RNA was analyzed using a NanoDrop 2000 Spectrophotometer (Thermo Scientific, catalog no. ND‐2000) to determine purity (260/280) and concentration (260 nm). Novogene (Sacramento CA) performed Human mRNA Sequencing (WBI), which included RNA sample quality control, mRNA library preparation (poly A enrichment), NovaSeq PE150 (6 G raw data per sample), Data quality control, and Standard Data Analysis, which generated data and figures presented in this text. Novogene was unaware of the identity of each sample. PCA was performed by Log_2_‐transforming FPKM values, calculating the standard deviation, and then centering the data by subtracting the mean and dividing by the standard deviation for each gene. PCA was utilized for data screening, which identified one outlier. This sample was repeated. The R‐package DESeq2 analysis was performed to determine differentially expressed genes. Two‐way clustering heatmaps were then generated using the R‐package pheatmap. Subclustering figures represent h‐clustering. Volcano plots display the Log_2_(Fold Change) on the *x*‐axis and the −Log_10_(*p*_adj_) on the *y*‐axis, where *p*_adj_ is an adjusted *p*‐value generated from a *t*‐test used to identify significantly differentially expressed genes between groups and/or samples.

## Conflict of Interest

The authors declare no conflict of interest.

## Author Contributions

B.M.R. conceived the study. E.B.S., B.M.R., and S.S. planned the experiments. E.B.S., S.S., K.R.S., K.K., and A.N.I. collected the data. E.B.S. and B.M.R. wrote the manuscript.

## Inclusion and Ethics

All individuals that contributed significantly to the manuscript were included as authors. The authors comply with the research inclusions and ethics policies of Boston University.

## Supporting information



Supporting Information

## Data Availability

RNA sequencing raw data files, raw counts, and fpkm values have been uploaded to Gene Expression Omnibus (GEO). Accession code is GSE281152. Any other raw data in the manuscript is available on request to the corresponding author.
