# Supplementary material for: Nanoplastics Enhance Transmembrane Transport and Uptake of Carcinogens: Transcriptional Changes and the Effects of Weathering
Source: Adv Sci (Weinh). 2025 Jun 20;12(31):e07355. doi: 10.1002/advs.202507355 (PMC12376499; doi:10.1002/advs.202507355)
Supplement: Supplementary file 1 — Supporting Information [file ADVS-12-e07355-s001.docx]

Supporting Information

**Nanoplastics Enhance Transmembrane Transport and Uptake of Carcinogens: Transcriptional Changes and the Effects of Weathering**

*Erik B. Schiferle ^1^, Saatwik Suman ^2^, Katherine R. Steffen ^2^, Koustav Kundu ^2^, Aniqa N. Islam ^2^, and Björn M. Reinhard ^1*^*

^1^ Division of Materials Science and Engineering, Department of Chemistry, and The Photonics Center, Boston University, Boston, MA, 02215, USA

^2^ Department of Chemistry and The Photonics Center, Boston University, Boston, MA, 02215, USA

^*^ bmr@bu.edu

**Supporting Figures**


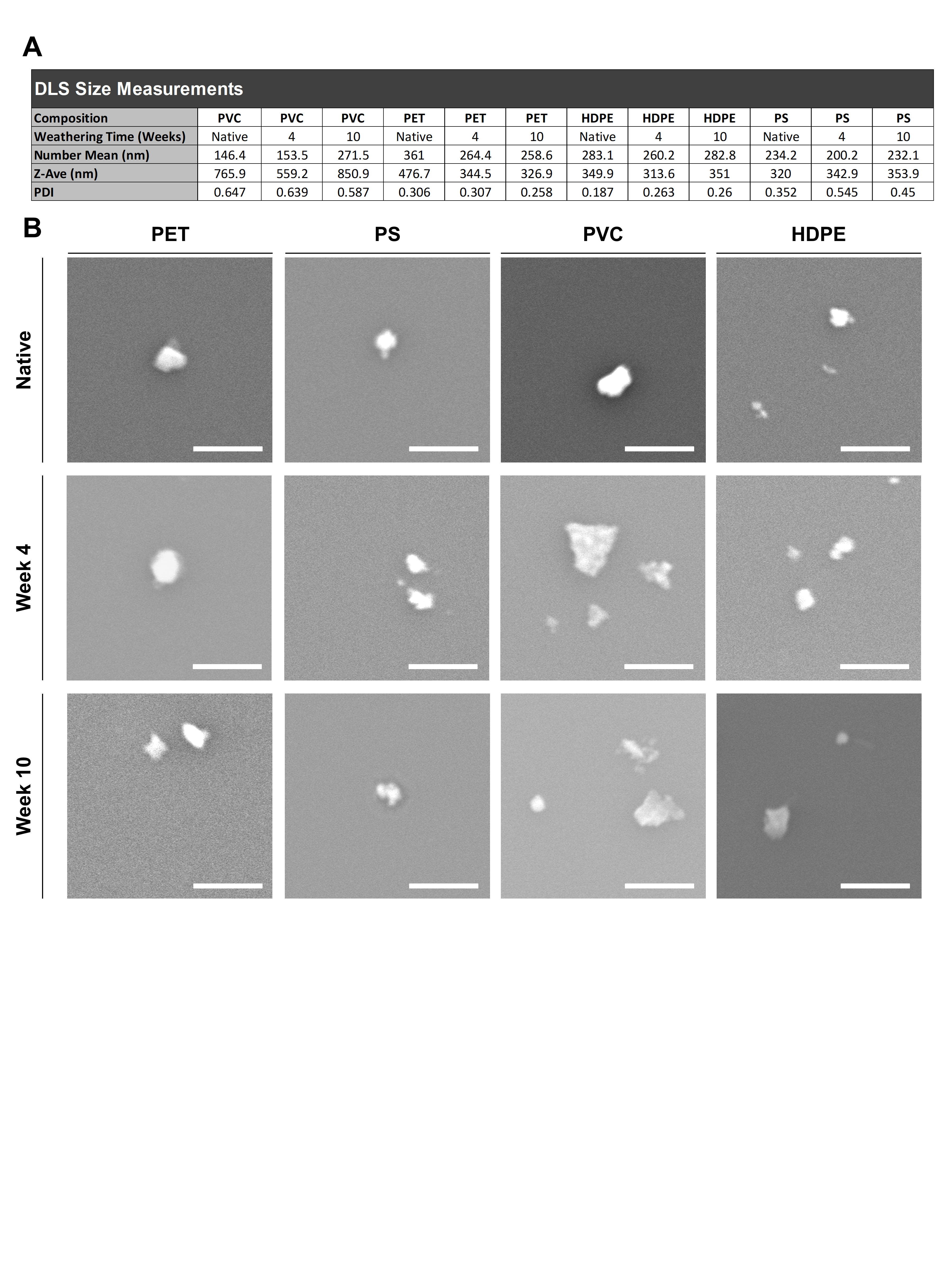


**Figure S1. Nanoplastics size and morphology.**

1. Z-average (nm), Number mean (nm), and Polydispersity Index (PDI) of the hydrodynamic diameter (nm) determined by dynamic light scattering (DLS) for native as well as 4 and 10 week weathered nanoplastics.
2. Representative SEM images of native as well as 4 and 10 week weathered nanoplastics for compositions PET, PS, PVC, HDPE. Magnification 35k×, 10kV, Scale Bar = 1µm.


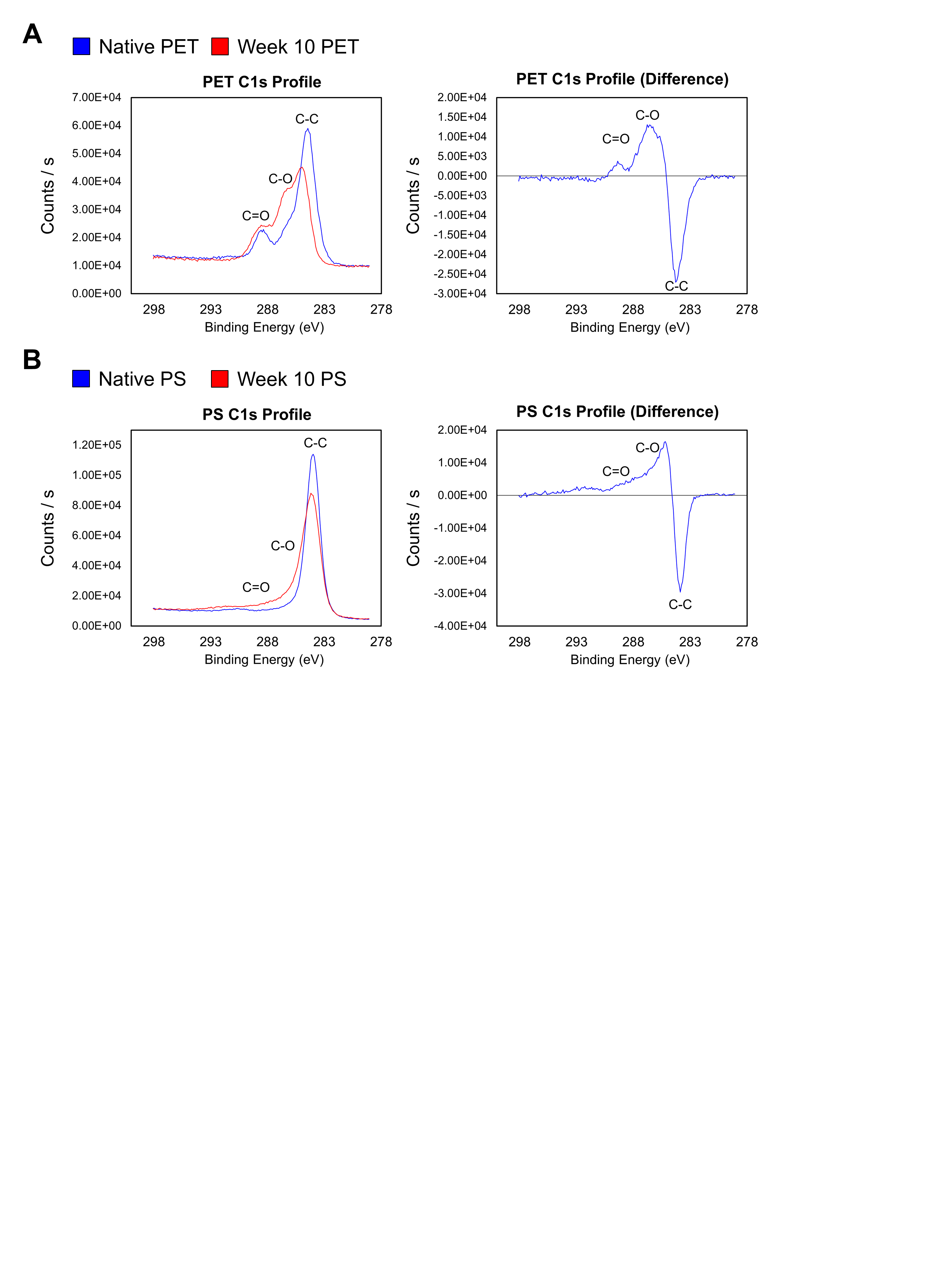


**Figure S2. X-ray Photoelectron Spectroscopy (XPS) of PET and PS.**

1. PET C1s XPS profile of native and week 10 nanoplastics for Counts/s vs. Binding Energy (eV) (left), and the subtraction of native from week 10 (right).
2. PS C1s XPS profile of native and week 10 nanoplastics for Counts/s vs. Binding Energy (eV) (left), and the subtraction of native from week 10 (right).


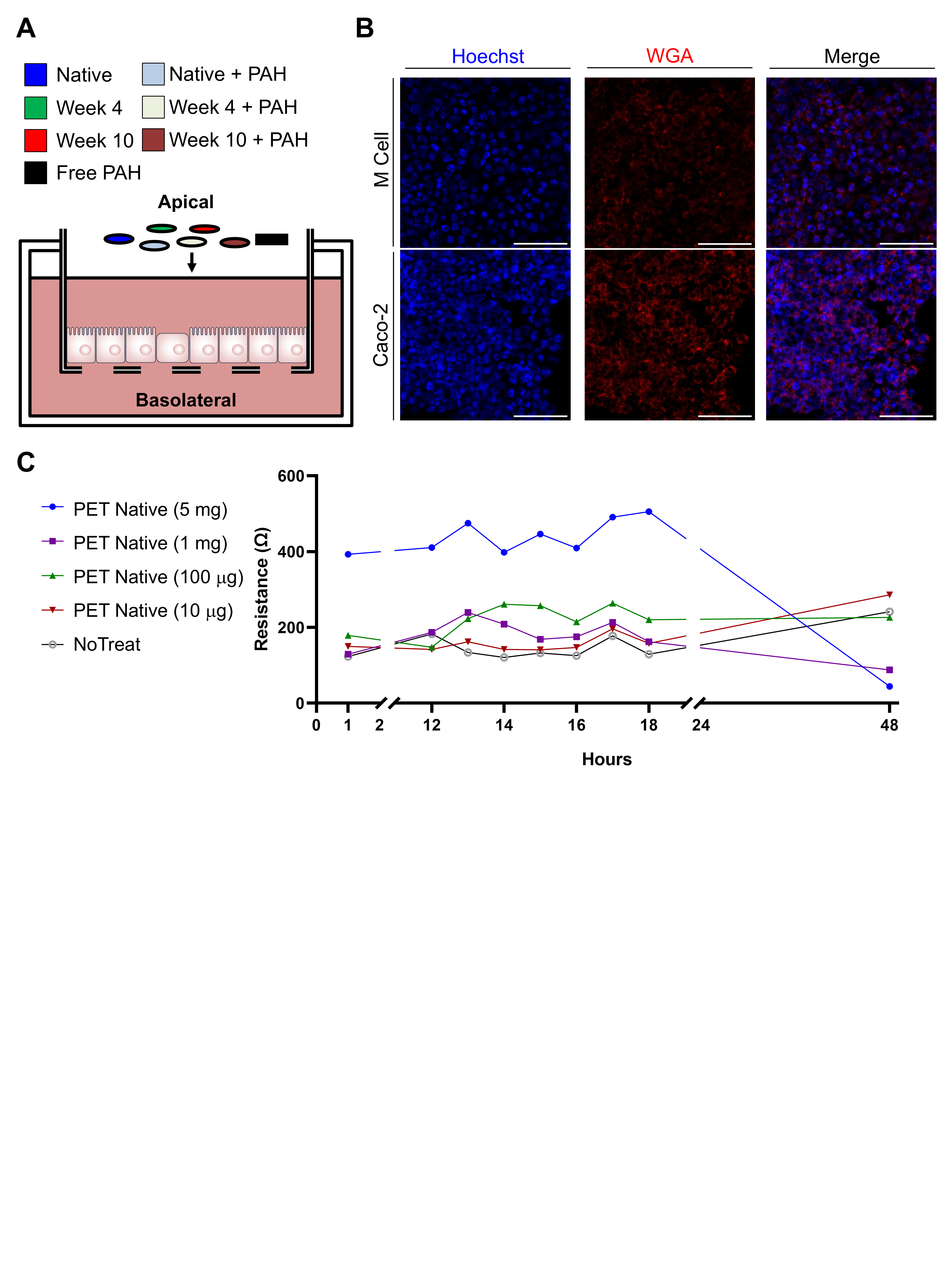


**Figure S3. Validating membrane integrity and establishing nanoplastics working concentrations.**

1. Schematic of the transwell insert containing the Caco-2/Raji B co-culture model (M cell model). Nanoplastics and PAHs can be added to the apical chamber in order to model the ability of the materials to traverse across the cell membrane, through the semi-permeable membrane, into the basolateral chamber.
2. Confocal fluorescence images of the M cell model and caco-2 cell model, stained with Hoechst (blue), WGA (red), and merged images. Images correspond to the M cell model and Caco-2 cell model images at depicted at 0 hours (before the addition of nanoplastics) in Figure 3C. Scale Bar = 100µm.
3. TEER measurements from M cell model transwell inserts. Unexposed cells (NoTreat), and cells exposed to 5 mg, 1 mg, 100 µg, or 10 µg of native PET nanoplastics monitored over the course of 48 hours.


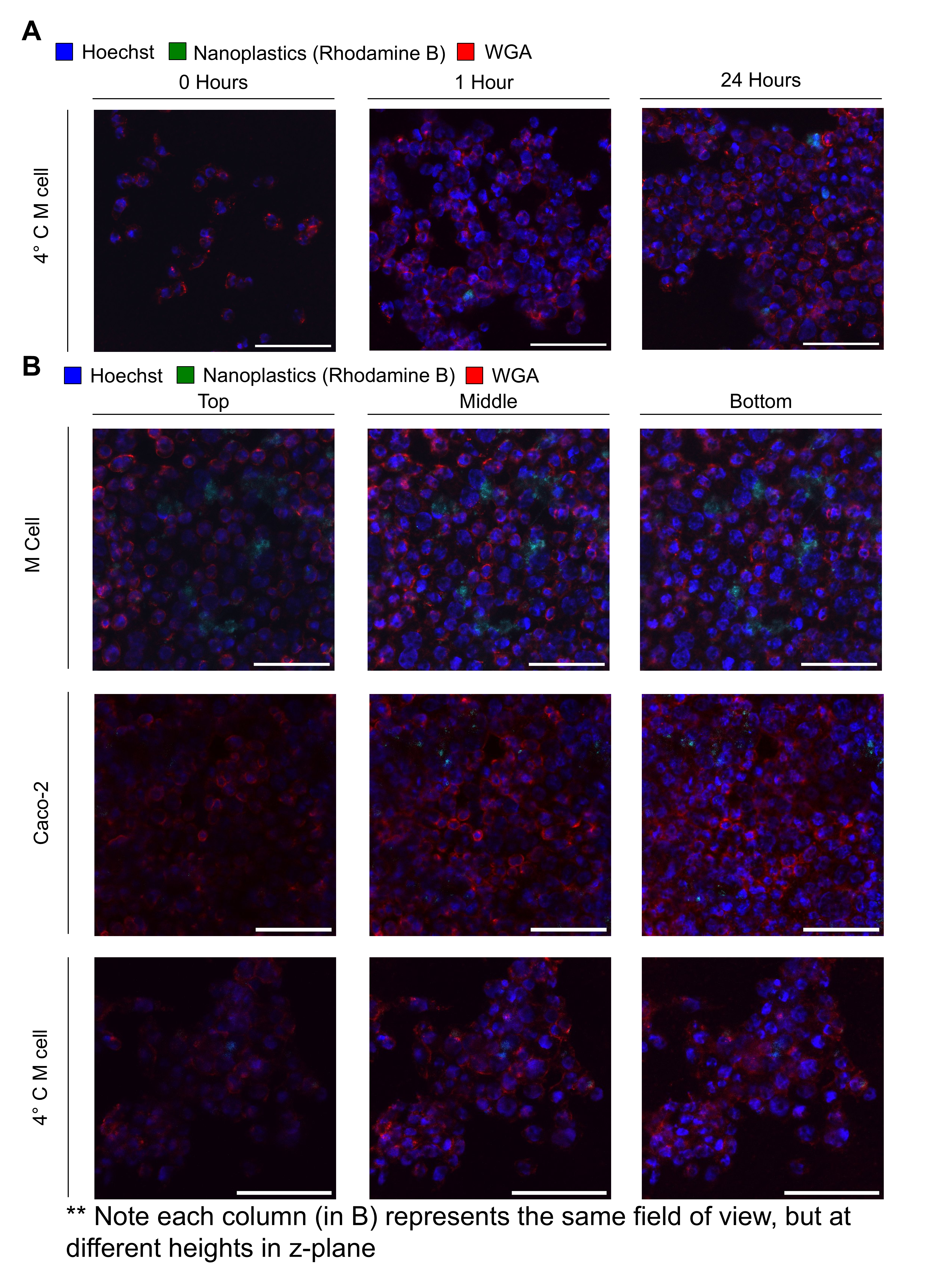


**Figure S4. Rhodamine-stained nanoplastics transport.**

1. Confocal fluorescence images of the M cell model at 4°C before addition of nanoplastics at 0 h as well as 1 h and 24 h after the addition of nanoplastics. The cells were stained with Hoechst (blue), WGA (red), and nanoplastics with rhodamine B (green). Scale bar: 100 µm.
2. Confocal fluorescence images of the M cell model, Caco-2 cells, and M cell model at 4°C after 24 h following the addition of rhodamine B nanoplastics. Images of the same field of view were taken in the apical plane (top), intracellular (middle), and basolateral plane (bottom). The cells were stained with Hoechst (blue), WGA (red), and nanoplastics with rhodamine B (green). Scale bar: 100 µm.


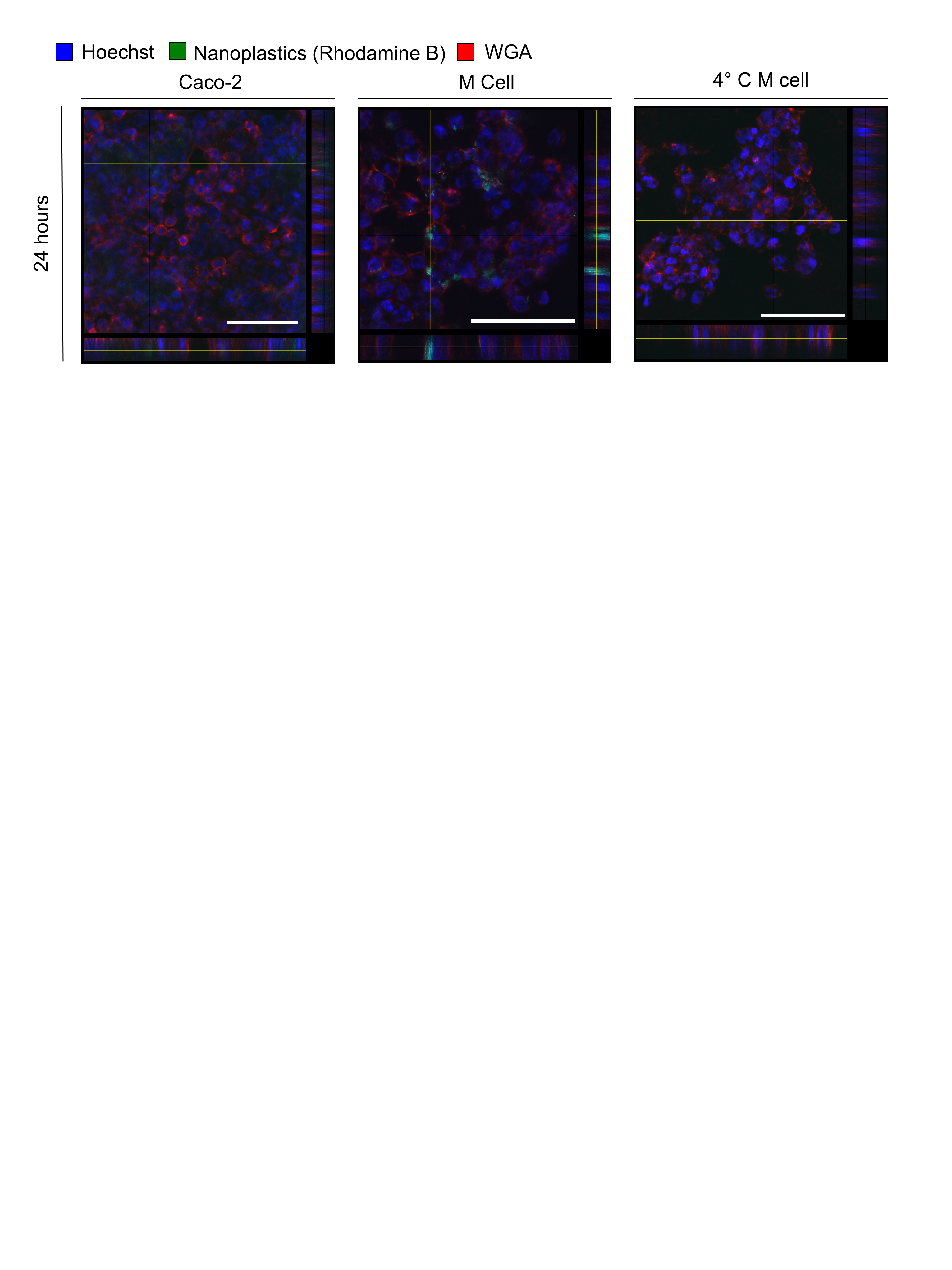


**Figure S5. Orthogonal transport images of Rhodamine-stained Nanoplastics.**

Confocal fluorescence images of (left to right): Caco-2 cell model, M cell model, M cell model at 4°C at 24 h after addition of rhodamine B-stained nanoplastics. The view of the x,y plane is augmented by cross-sectional views along the z-axis for the indicated lines. Cross-sections are shown on the bottom and right. Caco-2 only model and M cell model at 4°C images are taken from the same field of view as images in Figure S4B, and the M cell model image is a new field of view. The cells were stained with Hoechst (blue), WGA (red), and nanoplastics with rhodamine B (green). Scale bar: 100 µm.


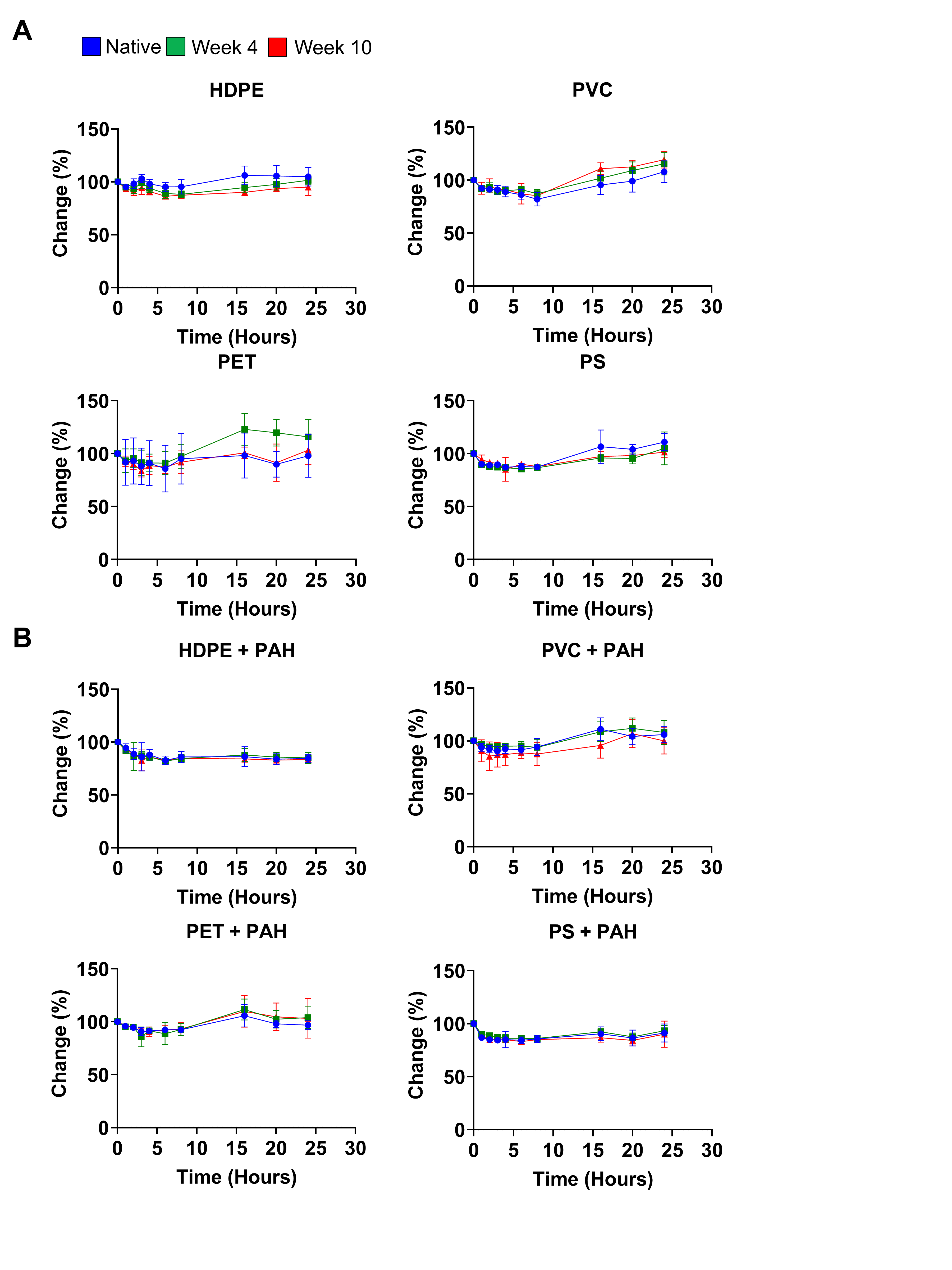


**Figure S6. TEER Measurements of M cell model cells exposed to nanoplastics.**

1. TEER measurements taken of M cell model cells exposed to native, Week 4, and Week 10 nanoplastics composed of HDPE, PVC, PET, PS taken over 24 hours. Error bars represent the mean percent change relative to NoTreat membrane cells ± standard deviation (SD, n=6 biological replicates).
2. TEER measurements taken of M cell model cells exposed to native, Week 4, and Week 10 nanoplastics composed of HDPE, PVC, PET, PS loaded with PAHs taken over 24 hours. Error bars represent the mean percent change relative to NoTreat membrane cells ± SD (n=6 biological replicates).


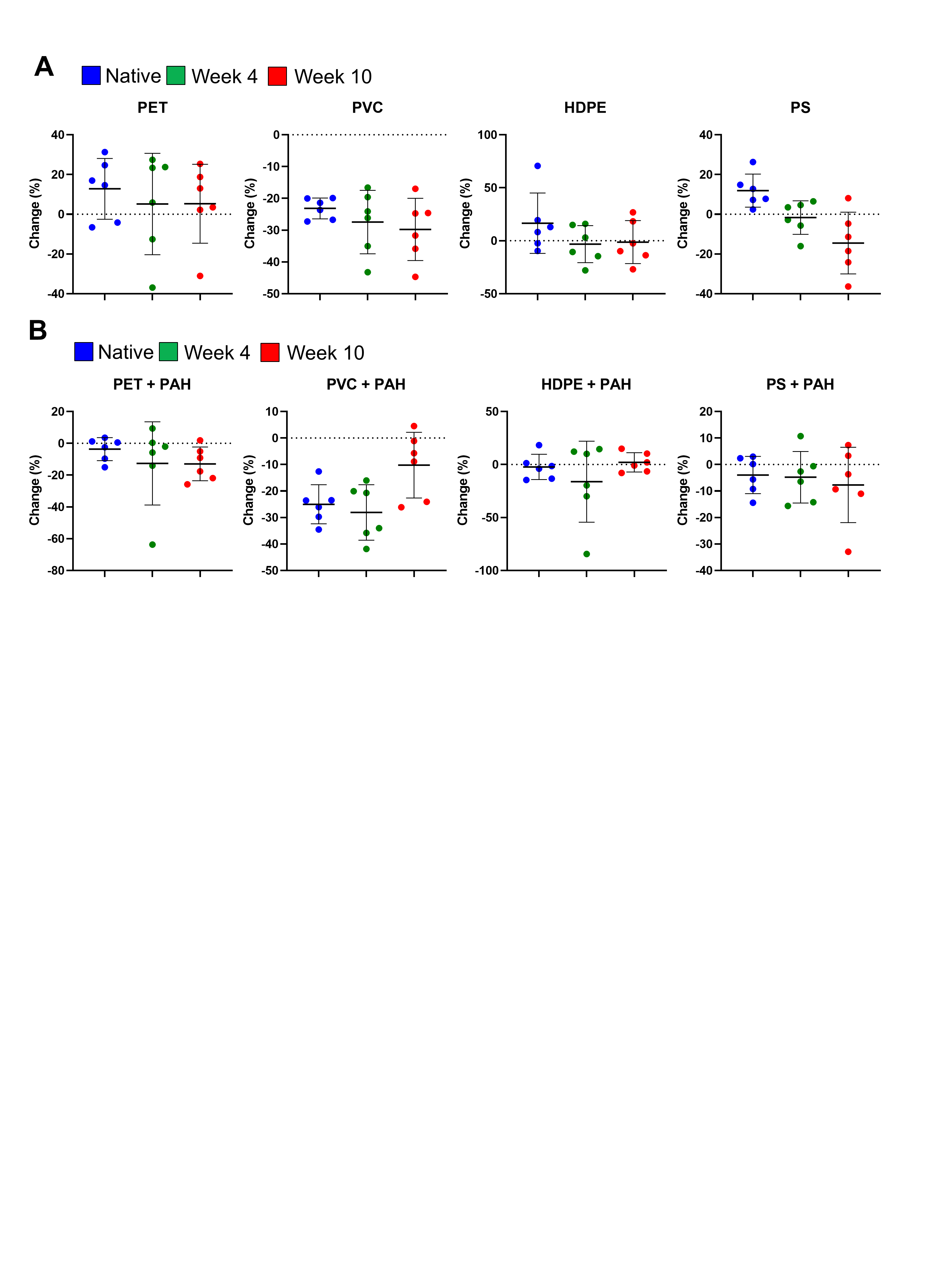


**Figure S7. LDH Measurements of M cell model cells exposed to nanoplastics.**

1. LDH measurements taken of M cell model cells exposed to native, week 4, and week 10 nanoplastics composed of HDPE, PVC, PET, PS for 24 hours. The percent change is relative to NoTreat cells. Error bars represent the mean percent change relative to NoTreat cells ± SD (n=6 biological replicates).
2. LDH measurements taken of M cell model cells exposed to native, week 4, and week 10 nanoplastics composed of HDPE, PVC, PET, PS loaded with PAHs for 24 hours. The percent change is relative to NoTreat cells. Error bars represent the mean percent change relative to NoTreat cells ± SD (n=6 biological replicates).


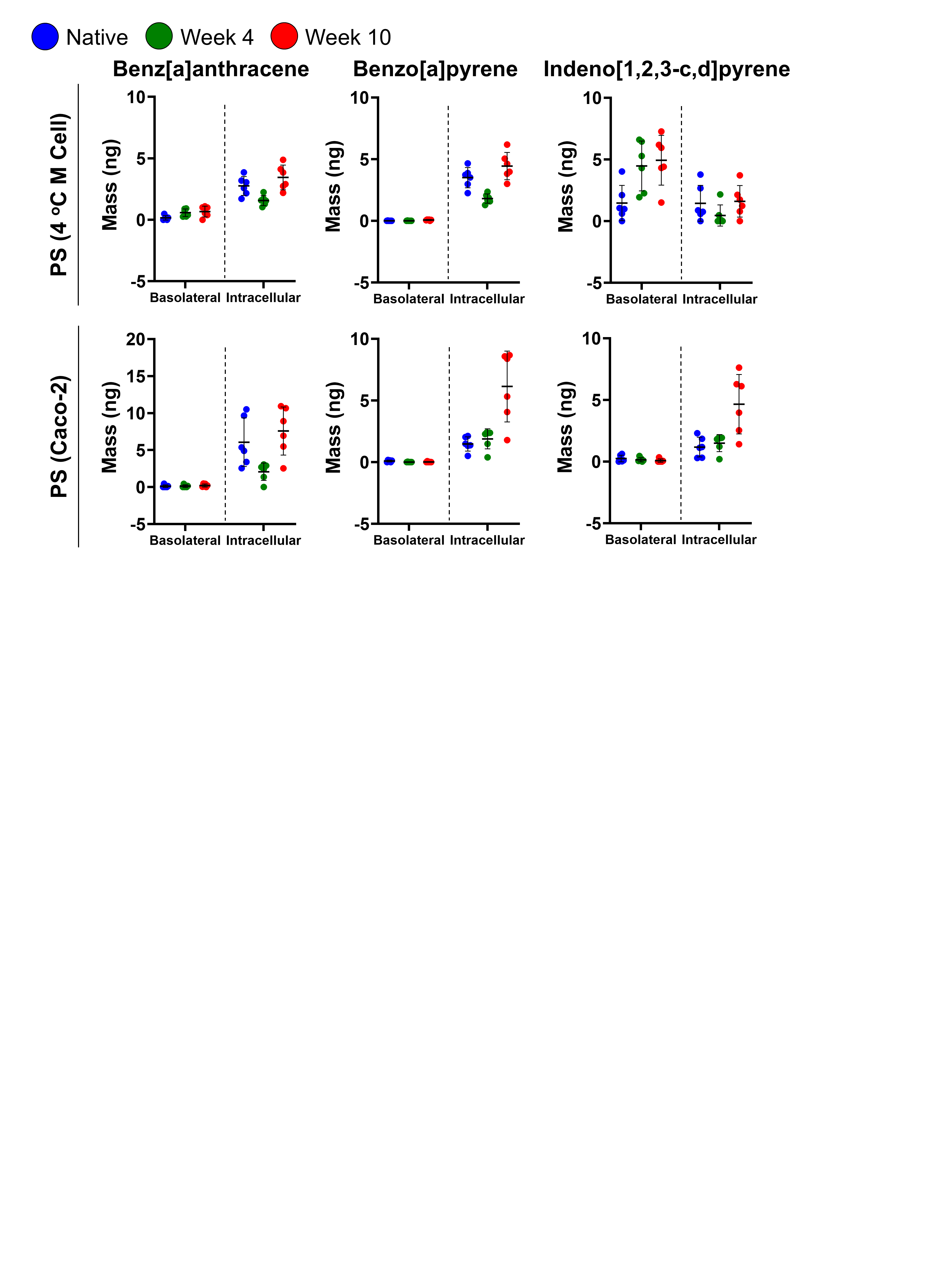


**Figure S8. PAH-loaded nanoplastics transport.**

Dot plots depict the mass (ng) of PAHs from PAH-loaded nanoplastics that traversed across the M cell model cells at 4°C or the caco-2 only cells (‘Basolateral’) or were taken up into cells (‘Intracellular’) for native, and 4 and 10 weeks weathered PS nanoplastics. Error bars represent the mean mass, baseline corrected by NoTreat cells ± SD (n=6 biological replicates).


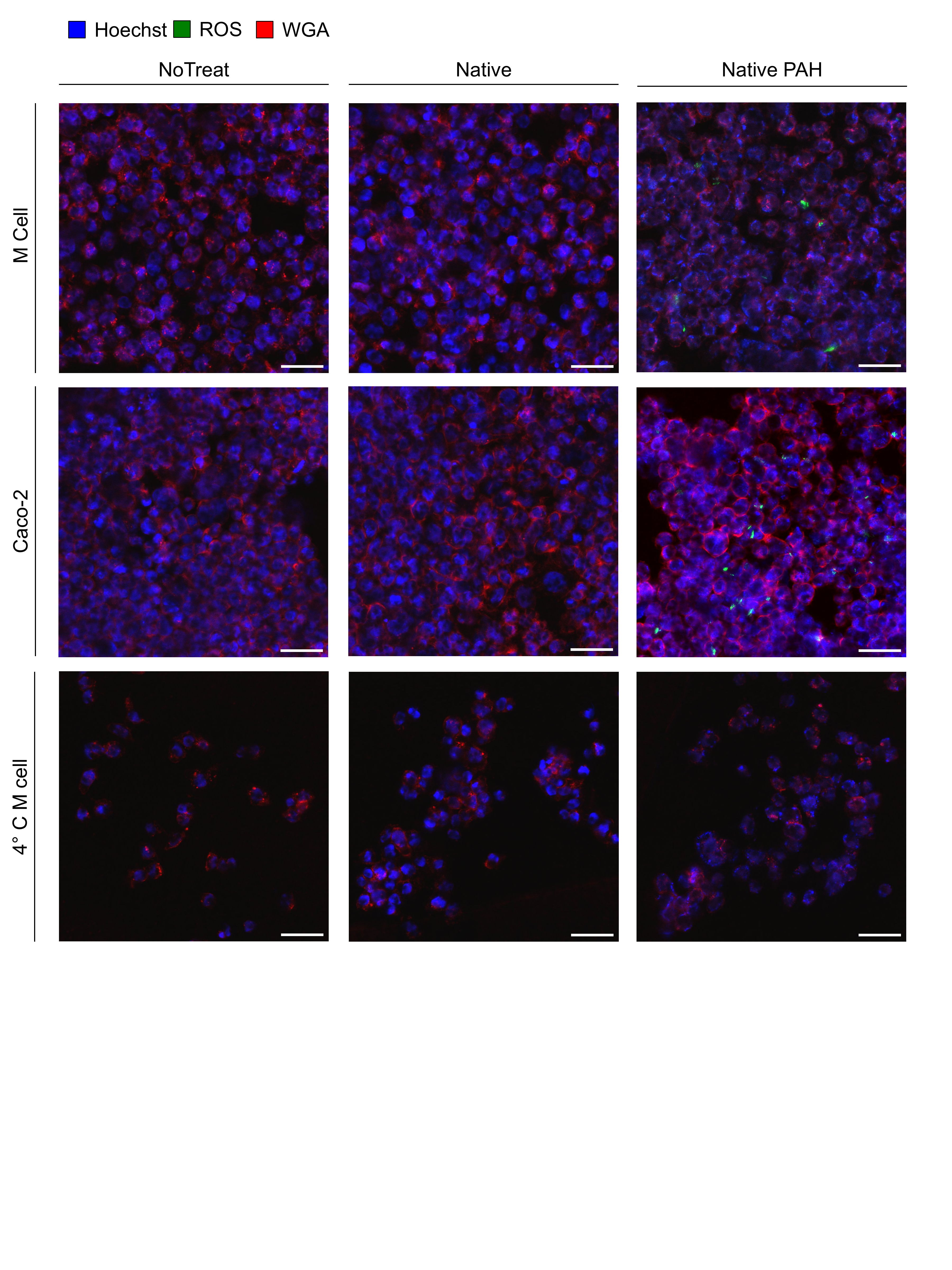


**Figure S9. ROS staining.**

Confocal fluorescence images taken of the M cell model, the Caco-2 model, and the M cell model at 4°C following treatment with an ROS staining kit that expresses green color under stress conditions. Images were taken of NoTreat cells, of cells treated with native PET nanoplastics, and native + PAH PET nanoplastics after 24 h of exposure. The cells were also stained with Hoechst (blue) and WGA (red). Scale bar: 50 µm.


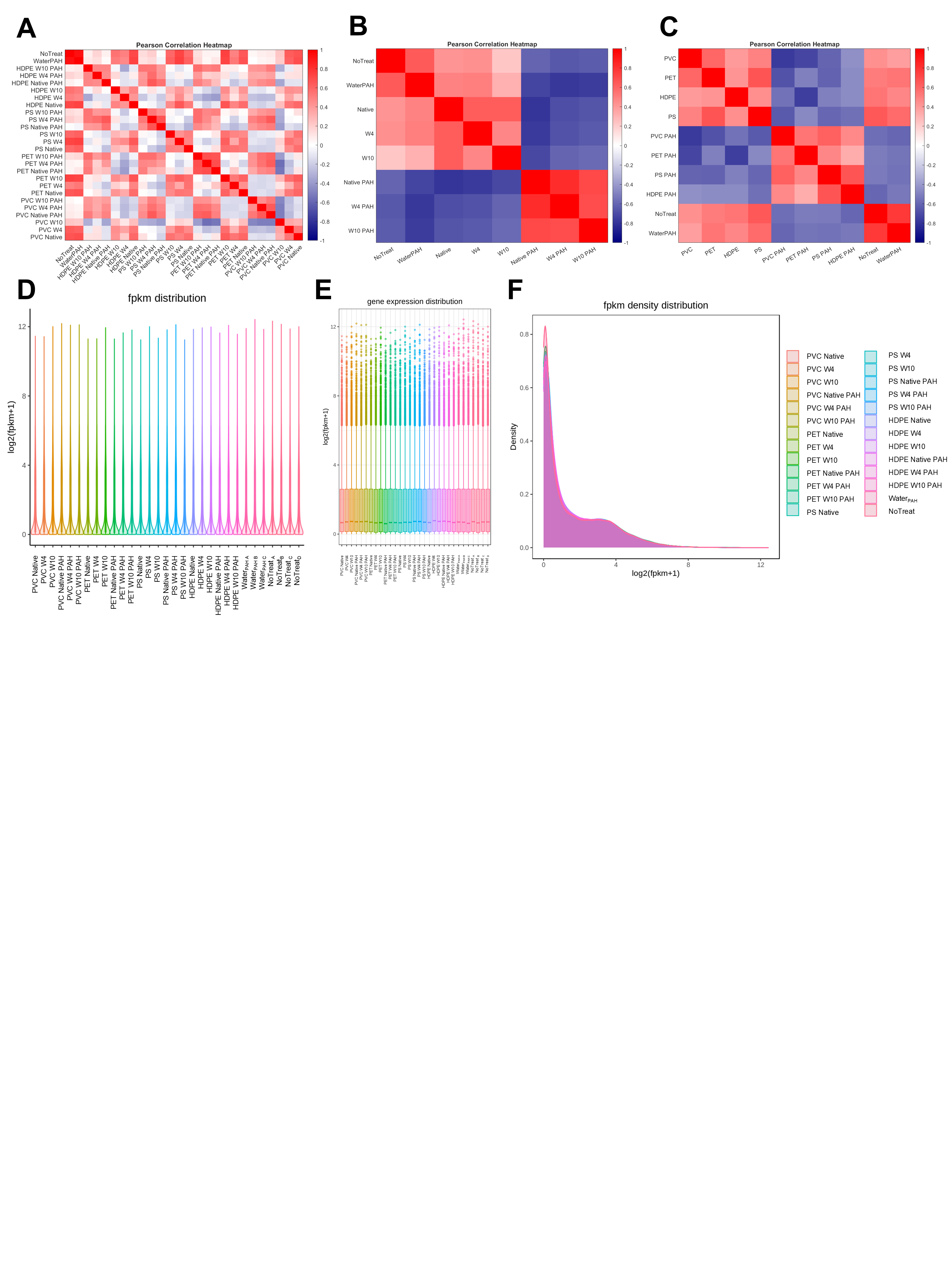


**Figure S10. Transcriptomic sample associations.**

**A-C)** Pearson Correlation plot for A) native, week (W4) and week 10 (W10) nanoplastics

composed of PVC, PET, PS, or HDPE with and without PAH loading as well as Water_PAH_ and

NoTreat controls s, B) samples grouped by composition HDPE, PVC, PS, PET with or

without PAH loading as well as Water_PAH_ and NoTreat controls, C) samples grouped by

weathering time (native, 4 weeks weathering, and 10 weeks weathering) with or without PAH

loading as well as the Water_PAH_ and NoTreat controls.

**D-F)** Violin plot (maxima, box depicts middle quartiles, bolded line represents the

Median, violin width depicts relative gene expression), box plot (maxima/outliers, box depicts

middle quartiles, bolded line represents the median), and fpkm density distribution plot of

native, week (W4) and week 10 (W10) nanoplastics composed of PVC, PET, PS, or HDPE

with or without PAH loading as well as Water_PAH_ and NoTreat controls. Individual biological

replicates of Water_PAH_ and NoTreat controls depicted to highlight reproducibility.


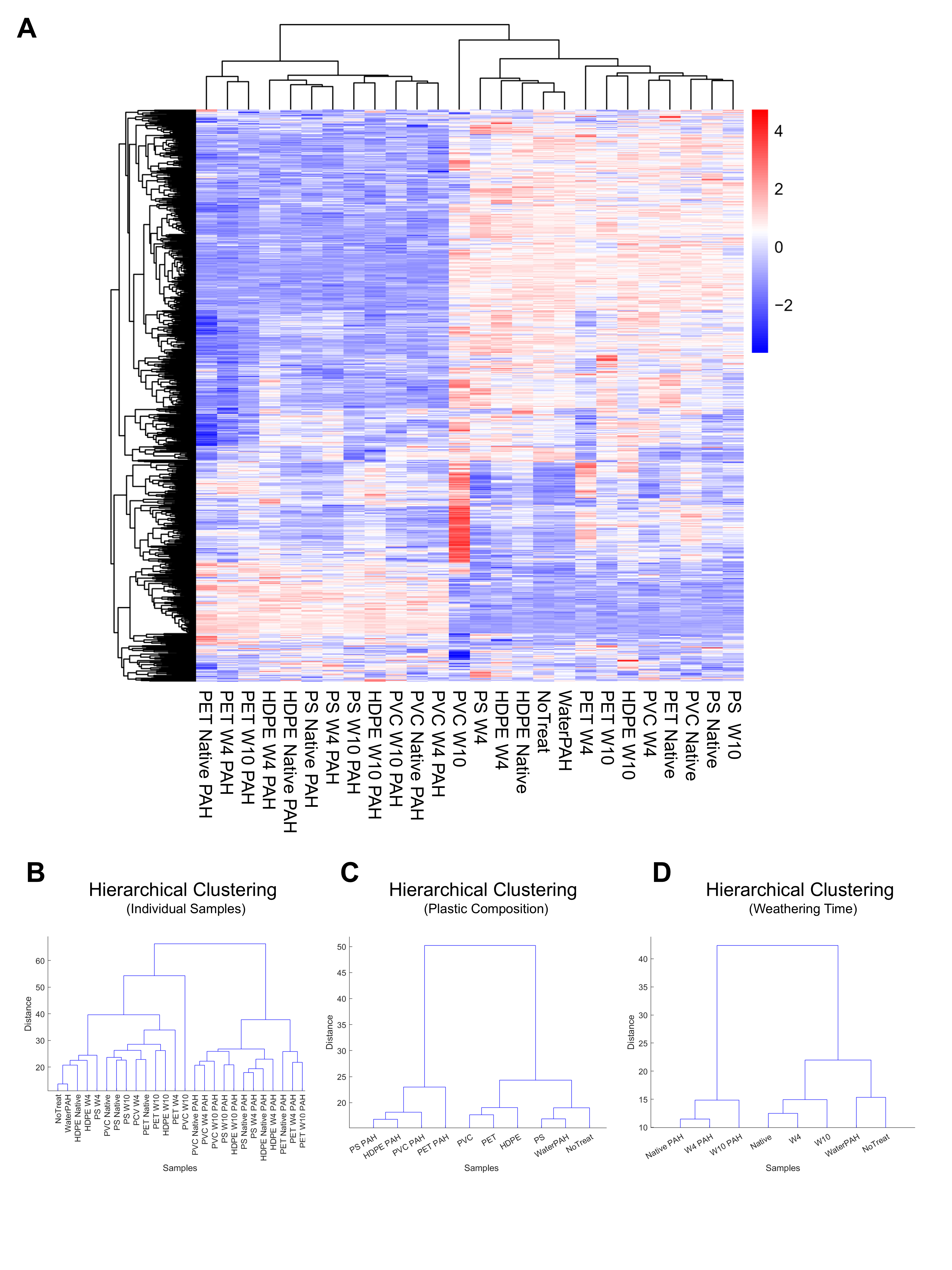


**Figure S11. Hierarchical clustering of DEGs.**

1. Two-way hierarchical clustering heat map of RNA-Seq transcriptome analysis of the DEGs

(712 total genes identified relative to NoTreat control, |Log_2_(fold change)| >= 1 and p_adj_ <=

0.05) for native, W4, and W10 nanoplastics composed of HDPE, PVC, PET, or PS with or

without sorbed PAHs, as well as Water_PAH_ and NoTreat control. X-axis represents conditions,

where y-axis represents genes. Data Log_2_ normalized.

**B-D)** Hierarchical clustering of samples for the same groups as **Figure S10 A-C**. Associations

measured by Euclidean distance after Log_10_-transform and normalization.


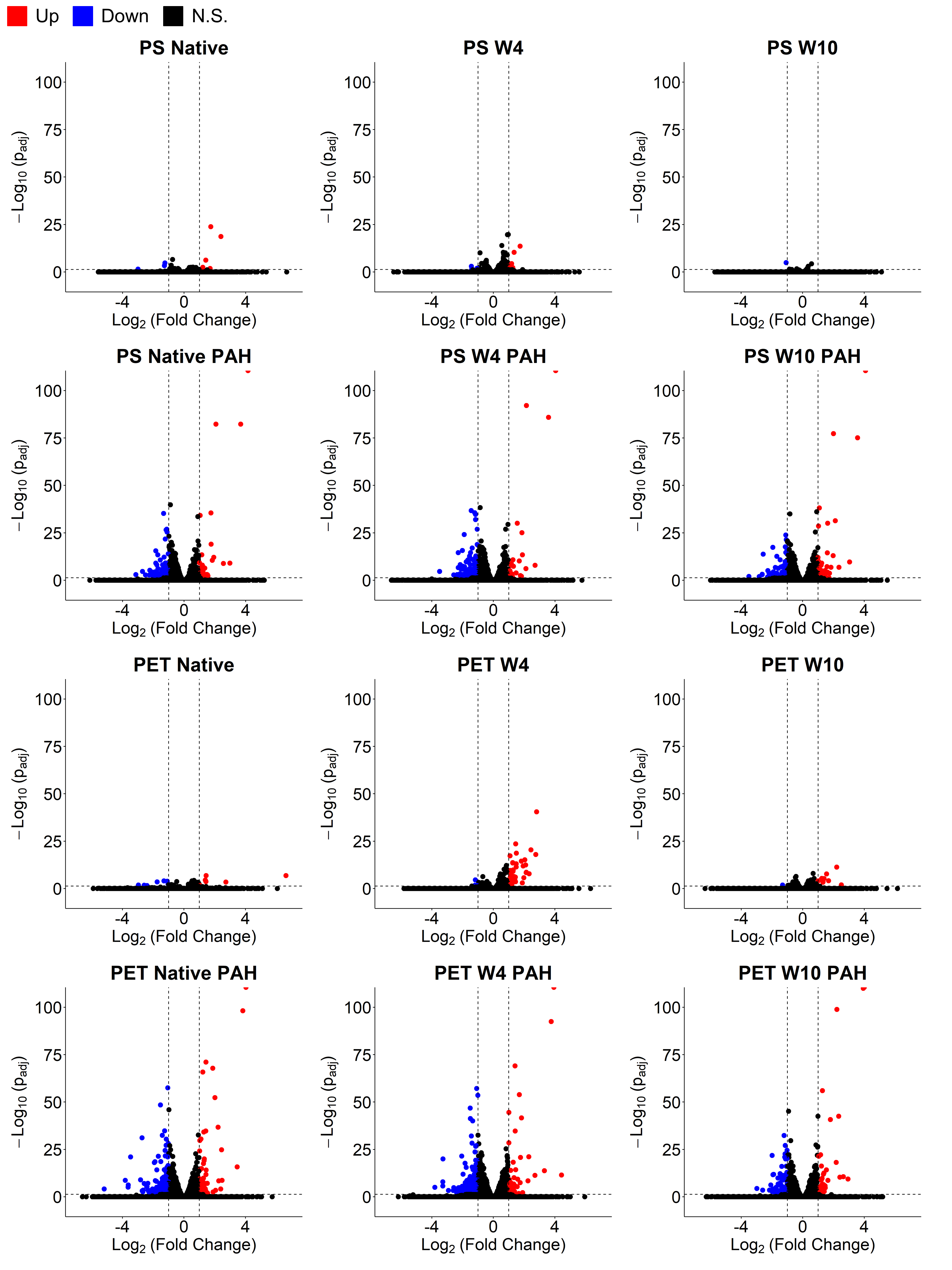


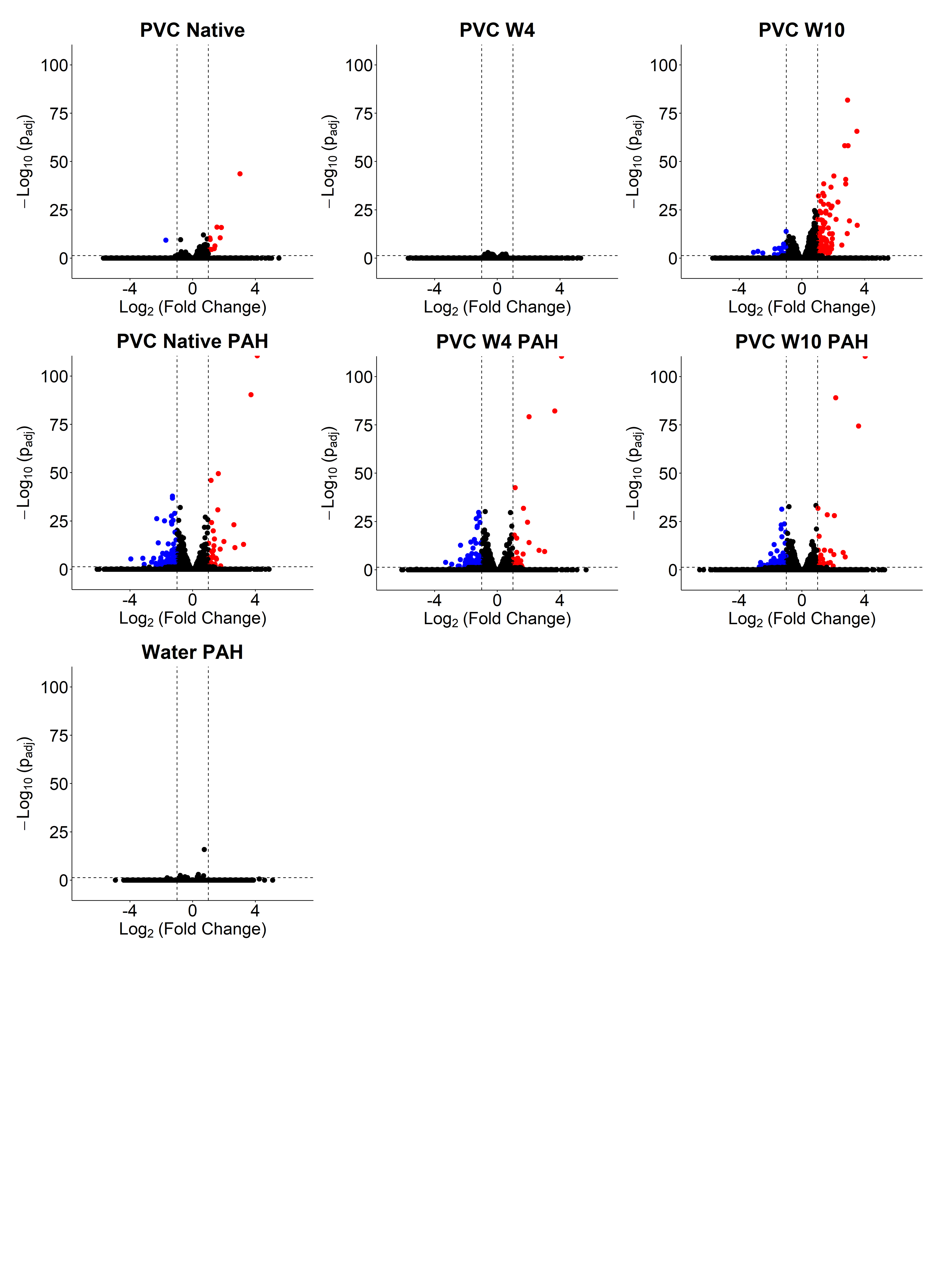


**Figure S12. Volcano Plots of DEGs.**

Volcano Plots of DEGs (712 total genes identified relative to NoTreat control, |Log_2_(Fold Change)| >= 1 and p_adj_ <= 0.05) for native, W4, and W10 nanoplastics composed of PVC, PET, or PS with or without sorbed PAHs, and Water_PAH_ samples. X-axis represents Log_2_(Fold Change) in gene expression, while y-axis represents -Log_10_(p-adjusted). Dashed lines correspond to values |Log_2_(fold change)| >= 1 and p_adj_ <= 0.05. Red dots represent genes above this threshold in the positive direction (Up), blue dots above this threshold in the negative direction (Down), black dots do not meet the criteria (N.S.).


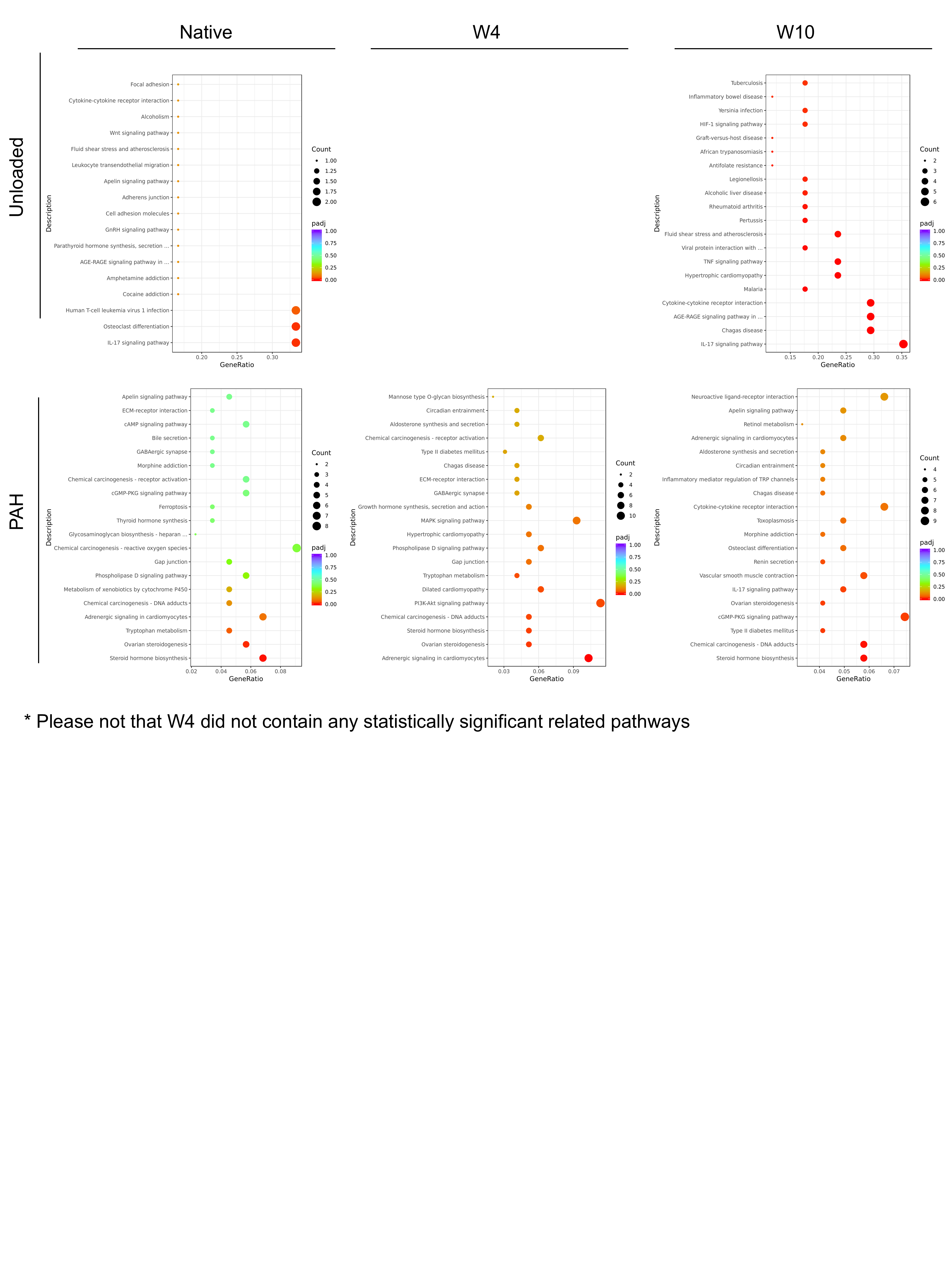


**Figure S13. KEGG pathway enrichment analysis.**

KEGG pathway enrichment analysis for samples grouped as native, nanoplastics weathered for 4 (W4) or 10 (W10) weeks with or without PAH loading.


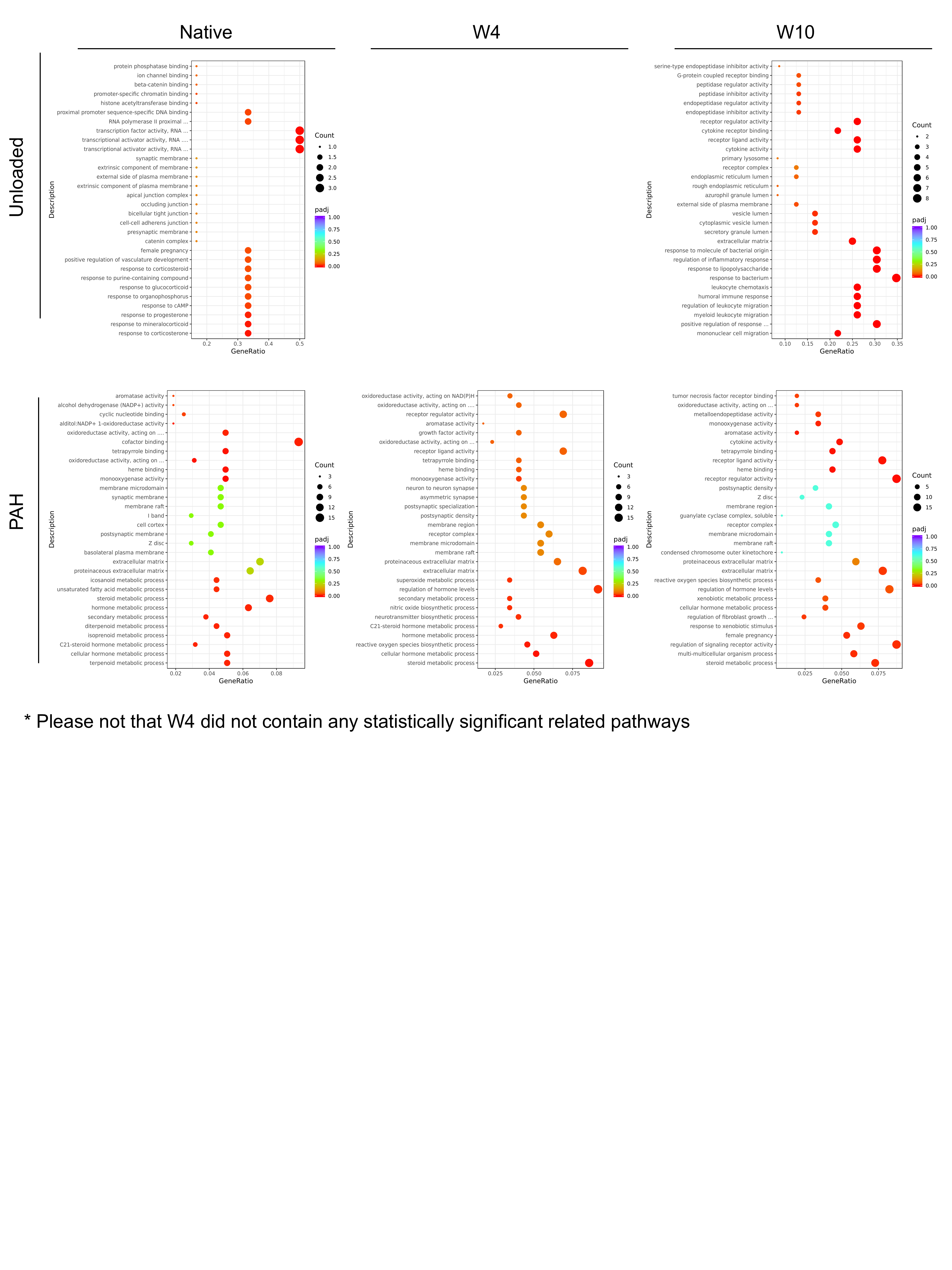


**Figure S14. GO pathway enrichment analysis.**

GO pathway enrichment analysis for samples grouped as native, nanoplastics weathered for 4 (W4) or 10 (W10) weeks with or without PAH loading.

**Supporting Tables**

**Supplementary Data Table** **1. Tabulated quantification of PAH sorption.**

PAH concentration in μg per mg of nanoplastics for native, 4 weeks weathered, or 10 weeks weathered PVC, PET, PS, HDPE nanoplastics (n = 8 replicates).
